# Factors related to irritable bowel syndrome and differences among subtypes: A cross-sectional study in the UK Biobank

**DOI:** 10.3389/fphar.2022.905564

**Published:** 2022-08-26

**Authors:** Kexin Wang, Huan Liu, Jingjing Liu, Liyuan Han, Zheng Kang, Libo Liang, Shengchao Jiang, Nan Meng, Peiwen Chen, Qiao Xu, Qunhong Wu, Yanhua Hao

**Affiliations:** ^1^ Department of Health Policy, School of Health Management, Harbin Medical University, Harbin, Heilongjiang, China; ^2^ Department of Social Medicine, School of Public Health, Harbin Medical University, Harbin, Heilongjiang, China; ^3^ Department of Epidemiology, School of Public Health, Harbin Medical University, Harbin, Heilongjiang, China; ^4^ Department of Global Health, Ningbo Institute of Life and Health Industry, University of Chinese Acadeny of Sciences, Ningbo, Zhejiang, China

**Keywords:** disorders of gut–brain interaction, irritable bowel syndrome, Rome Ⅲ, prevalence, risk factors, gender, subtypes

## Abstract

**Background:** Irritable bowel syndrome (IBS) reduces patients’ quality of life and causes great burdens due to its unclear pathogenesis and criteria for diagnosis. This study aimed to explore the differences in prevalence and the influencing factors for IBS and its subtypes.

**Methods:** The UK Biobank surveyed 174,771 adult participants who completed the Digestive Health Questionnaire (DHQ) through emails and websites. DHQ included the Rome III criteria, IBS symptom severity score, and Patient Health Questionnaire 12 Somatic Symptom score. The UK Biobank also asked regarding previous IBS diagnosis, diagnosis for post-infectious IBS (PI-IBS), and environmental exposures and associated conditions (including anxiety or depression, based on treatment sought or offered). Pearson’s Chi-squared test or Wilcoxon’s rank-sum test was used for potential associations. Binary logic regression based on sex stratification was used to examine associations between selected factors and IBS and its subtypes.

**Results:** This study included 31,918 participants who met the Rome III criteria for IBS. The pooled prevalence of IBS in the UK Biobank was 18.3%, with mixed IBS as the predominant subtype (59.0%), followed by diarrhea-predominant IBS (25.1%), constipation-predominant IBS (14.7%), and untyped IBS (1.1%). IBS was significantly associated with somatization (male: OR = 5.326, 95% CI = 4.863–5.832; female: OR = 4.738, 95% CI = 4.498–4.992) and coeliac disease (male: OR = 4.107, 95% CI = 3.132–5.385; female: OR = 3.783, 95% CI = 3.310–4.323). Differences in antibiotics and mental status were presented among subtypes and sex. Furthermore, 1,787 individuals were diagnosed with PI-IBS in the group of patients with IBS. The prevalence of PI-IBS in IBS was 16.6% in the UK Biobank, and it was characterized by diarrhea, fever, bloody diarrhea, and vomiting.

**Conclusion:** Somatization and coeliac disease are primary risk factors for IBS. Distinguishing differential risk factors is critical for the precise diagnosis and treatment of IBS subtypes, particularly sex-specific differences in mental health status. General practitioners should focus on the treatment according to IBS subtypes.

## 1 Introduction

Irritable bowel syndrome (IBS), defined as the disorder of gut–brain interaction (DGBI), had pooled prevalence rates of 10.1% in countries based on an internet survey and 3.5% in countries based on a household survey, which used Rome Ⅲ diagnostic criteria (Sperber et al., 2021). Its characteristic symptoms include abdominal pain and altered bowel habits, including stool consistency and frequency (Drossman, 2006). Although the pain and disturbing symptoms caused by IBS are not life-threatening, they severely impair the quality of life of patients and result in tremendous economic burden (Wong and Drossman, 2010; Canavan et al., 2014a; Tack et al., 2019).

The obscure pathophysiology and lack of specific biomarkers for IBS make its diagnosis difficult (Fichna and Storr, 2012). In addition, many overlapping symptoms between IBS and other comorbidities, such as coeliac disease, increase the difficulty of the diagnosis of IBS (Aziz and Simrén, 2021). To date, the diagnosis of IBS is based on symptoms according to Rome criteria, in which the positive diagnosis for IBS was updated from Rome Ⅲ to Rome Ⅳ in 2016. The Rome IV criteria are more restrictive than the Rome Ⅲ criteria. For example, the global prevalence of IBS was 3.8% with the Rome IV criteria and 9.2% with Rome Ⅲ criteria (Oka et al., 2020). Aziz et al. (2018) showed a lack of major implications in the diagnosis of IBS from Rome Ⅲ to Rome Ⅳ, and patients with IBS diagnosed by Rome Ⅳ had more severe clinical symptoms.

Based on the current research, the diagnosis of IBS remains unelucidated; therefore, it is important to gain a fundamental understanding of the potential factors influencing IBS for better diagnosis and treatment. The female sex, younger age, and lower income were recognized as IBS risk factors (Drossman et al., 1993; Talley et al., 1995; Jiang et al., 2019). However, a recent study showed that the pooled prevalence of IBS was 11.5% between 18 and 39 years of age, 9.7% between 40 and 64 years of age, and 7.5% over 65 years of age (Sperber et al., 2021). This implies that patients with IBS aged over 40 years should be more concerned. Heredity may also be an important risk factor for IBS, with an incidence of approximately 33% (Whorwell et al., 1986). A cohort study showed that patients with IBS reported antibiotic use of 29.2%, with a 1.8-fold risk of IBS (Krogsgaard et al., 2018). Another study explored the possible cumulative effects of psychological changes on the severity of the gastrointestinal symptoms of IBS (Midenfjord et al., 2020). Symptoms of IBS included not only gastrointestinal symptoms but also extraintestinal symptoms (Whitehead et al., 2002; Patel et al., 2015). Therefore, some influencing factors related to symptoms of IBS caused general concern (Black et al., 2020). In the UK, Black et al. (2020) reported that somatization measured by the Patient Health Questionnaire 12 (PHQ-12) was independently associated with the severity of IBS symptoms. They proposed a process whereby gastrointestinal symptoms and discomfort caused severe IBS symptoms, which prompted patients to pay further attention to the symptoms of IBS. Moreover, some diseases with similar symptoms to IBS, such as coeliac disease, should be explored in the future (Ford et al., 2009). Common foods, including wheat, barley, and rye, (Baydoun et al., 2012), contain certain ingredients that may also trigger discomfort and symptoms, such as food intolerance, in patients with IBS (Eswaran et al., 2011; Böhn et al., 2013).

Based on the Rome Ⅲ bowel habit subclassification (Longstreth et al., 2006), IBS was classified into four subtypes: constipation-predominant IBS (IBS-C), diarrhea-predominant IBS (IBS-D), mixed IBS (IBS-M), and untyped IBS (IBS-U). However, only a few studies comprehensively focused on the differences among these IBS subtypes. Using psychological factors as an example, single-subtype IBS-M presented a higher level of depression and anxiety (Hu et al., 2021). Comparing IBS-C with IBS-D, the prevalence of anxiety and depression were markedly different (Fond et al., 2014). Overall, research works exploring the differences of potential risk factors among subtypes were limited.

Post-infectious irritable bowel syndrome (PI-IBS) may appear after acute gastroenteritis or following an episode of infective gastroenteritis as a special subtype of IBS (Dunlop et al., 2003), with a pooled prevalence varying from 7% to more than one-third of all IBS cases (Schwille-Kiuntke et al., 2011). Some studies reported the influencing factors for PI-IBS; however, differences in influencing factors for PI-IBS and general IBS subtypes remain unclear.

This cross-sectional study aims to examine the differences in the prevalence of IBS and the influencing factors associated with IBS and its subtypes based on the Rome III criteria using UK Biobank’s extensive sample data of adults aged 40–69 years in the United Kingdom. It also aims to provide guidance for better diagnosis and treatment of IBS in the United Kingdom and other nations.

## 2 Materials and methods

### 2.1 Participants

The UK Biobank was a large-scale multiple cohort study consisting of approximately 500,000 individuals (aged 40–69 years) recruited from across the United Kingdom between 2006 and 2010 (Collins, 2012). At the end of 2015, a team led by a group of gastroenterologists and the UK Biobank jointly planned and designed a survey on an extremely common abdominal disease, IBS, which was performed in 2017. Participants in the study were from the baseline survey and were invited mainly by email and on the participant website. As of 18 July 2018, about 174,771 participants completed the web-based questionnaire, including the participants’ self-reported socioeconomic information and the Rome III criteria, IBS symptom severity score (IBS-SSS), and PHQ-12 questionnaires. The UK Biobank received ethical approval from the National Health Service National Research Ethics Service 11/NW/0382 and was part of the UK Biobank project 52632.

### 2.2 Inclusion and exclusion criteria

In this study, IBS was diagnosed based on the Rome III questionnaire, and participants with missing data in the Rome III questionnaire were excluded. A total of 174,217 participants completed the Rome III questionnaire, including 31,918 participants with IBS and 142,299 participants with non-IBS; this data was used to calculate the prevalence of IBS in the UK Biobank. To further analyze the factors associated with IBS and its subtypes, this study included participants with moderate to severe IBS symptoms based on an IBS-SSS score of ≥ 175 (Bonfiglio et al., 2018). The exclusion criteria were as follows: 1) participants with IBS who had missing data in IBS-SSS, 2) participants with IBS who had no symptoms or with mild symptoms (IBS-SSS score of <175), and 3) participants who could not confirm a history of IBS. The exclusion criteria eliminated the effect of IBS history on factors associated with IBS and its subtypes. Finally, 147,336 participants were included in this study, in which 17,695 had IBS and 129,641 were without IBS (non-IBS).

**FIGURE 1:**
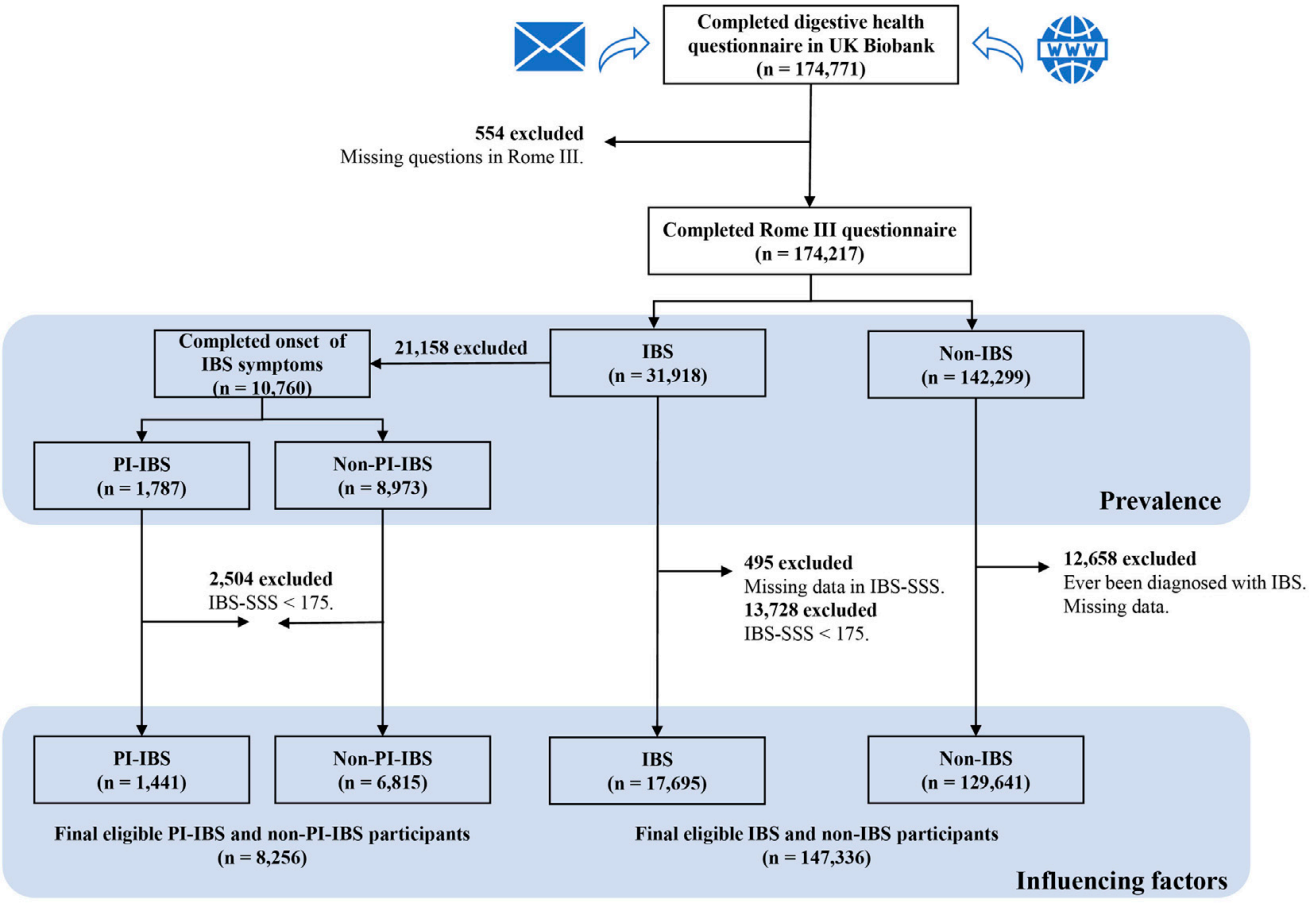
Flowchart of participant inclusion and exclusion. IBS, irritable bowel syndrome; non-IBS, participants without IBS; PI-IBS, post-infectious IBS; non-PI-IBS, participants without PI-IBS but with IBS; and IBS-SSS, IBS symptom severity score.

The UK Biobank used all IBS respondents who met the diagnosis of the Rome III criteria (*n* = 31,918) to identify PI-IBS further. In the UK Biobank database, only those who had been diagnosed with IBS (with IBS history) answered questions regarding the onset symptoms of IBS in the questionnaire. Therefore, only participants with a history of IBS diagnosis were included to define PI-IBS. Finally, a sample size of 10,760 participants were included, in which 1,787 were with PI-IBS and 8,973 were without PI-IBS but with IBS (non-PI-IBS), which was used to estimate the prevalence of PI-IBS among IBS patients in the UK Biobank. Moreover, this study also excluded the data with IBS-SSS score of < 175 to analyze the associations between PI-IBS and non-PI-IBS, which determined a sample size of 8,256 patients (Figure 1).

### 2.3 Study measurements

#### 2.3.1 Diagnosis of irritable bowel syndrome and its subtypes

The Rome III criteria were used to diagnose IBS based on symptoms such as chronic abdominal pain or discomfort at least 3 days per month in the last 3 months associated with two or more following symptoms: 1) improvement with defecation, 2) the onset of a change in the frequency of stool, and 3) the onset of a change in the form or appearance of stool. Moreover, the criteria included the last 3 months with symptom onset at least 6 months prior to diagnosis. The IBS subtypes were defined in terms of stool forms (hard/lumpy and loose/watery in at least 25% of evaluations).

#### 2.3.2 Diagnosis of post-infectious irritable bowel syndrome

Our study defined PI-IBS based on the criteria set in previous studies (Dunlop et al., 2003; Johnsen et al., 2018) as follows: 1) patients with a confirmed diagnosis of IBS, 2) patients with a sudden onset of IBS and also diagnosed with an infectious disease when the IBS symptoms first appeared (or 2 weeks prior), and 3) patients with two or more symptoms, including fever, diarrhea, bloody diarrhea, and vomiting.

#### 2.3.3 Irritable bowel syndrome symptoms

IBS-SSS was used to determine the severity of IBS symptoms experienced within the previous 3 months, including abdominal pain, distension, satisfaction with bowel habits, and interference with the participants’ life in general. IBS-SSS yielded a total score ranging from 0 to 500, and the scores were divided into four categories: remission of IBS symptoms (0–74), mild IBS (75–174), moderate IBS (175–299), and severe IBS (300–500) (Card et al., 2018).

#### 2.3.4 Extraintestinal somatic symptoms

The PHQ‐12 is a modified version of the commonly used PHQ-15, which is a validated questionnaire that assesses the severity of somatic symptoms (Francis et al., 1997). Participants were asked to rate the severity of 12 symptoms over the previous 3 months. These symptoms, one of which was only applicable to women, were rated from 0 (not bothered at all) to 2 (extremely bothered). Therefore, the total PHQ-12 score ranged from 0 to 24 for women and from 0 to 22 for men. In this study, the PHQ-12 score was used to identify whether participants experience somatization symptoms (Polster et al., 2018). A PHQ-12 score of > 6 was defined as high somatization, whereas a PHQ-12 score of ≤ 6 was defined as low somatization.

#### 2.3.5 Anxiety, depression, and other potential factors

Regarding mental health, the two main variables included anxiety and depression. Participants were asked the following questions: “Have you ever been offered or sought treatment for anxiety?” and “Have you ever been offered or sought treatment for depression?” Participants’ mode of birth was also asked as follows: “Were you born by caesarean section?” Furthermore, participants’ IBS family history was examined with the following question: “Do you have a family history of IBS in your parents/siblings/children?” We also considered previous antibiotic misuse, which was assessed with the question: “During childhood or as a teenager, did you receive long-term or recurrent courses (3 or more per year) of antibiotics (for example, for tonsillitis or acne)?” Other health issues that may affect IBS were also assessed. Participants were asked whether they had been diagnosed with coeliac disease or gluten sensitivity. After participants who selected “prefer not to answer” or “do not know” or “missing” were considered as “missing data,” the final response categories were included, with 1 = yes, 0 = no, and missing data.

#### 2.3.6 Demographic and socioeconomic variables

In this study, potential demographic and socioeconomic variables, including age, sex, and socioeconomic status (Townsend Deprivation Score), were analyzed. Age was determined by the baseline age and time when participants completed the Digestive Health Questionnaire. The Townsend Deprivation Score was calculated immediately prior to the participant joining the UK Biobank. Participants were assigned a score corresponding to the output area in which their postcode was located. The Townsend Deprivation Score was derived from the participants’ postcode, with negative scores reflecting relatively greater affluence (Spiller et al., 2010).

### 2.4 Statistical analysis

Descriptive statistics were calculated, including frequencies and percentages for binary categorical variables, and median and interquartile ranges were presented for continuous variables that were not normally distributed. A univariate analysis was performed using Pearson’s chi-squared test or Wilcoxon’s rank-sum test. Binary logic regression was used to examine associations between IBS and its associated factors. Multinomial logistic regression models were used to study the relationship between the independent variables and IBS subtypes (i.e., taking non-IBS patients as the reference standard). Data were stratified by sex to identify the differences in influencing factors of IBS. We adjusted for demographic and socioeconomic factors in Models 2 and 4, respectively. All data were analyzed by SAS V.9.4 (SAS Institute), and statistical significance was set at *p* < 0.05.

## 3 Results

### 3.1 Participants’ characteristics

There were 31,918 participants diagnosed with IBS by the Rome III criteria out of the 174,217 participants who completed the Rome III questionnaire; therefore, the estimated prevalence of IBS based on the Rome III criteria in the UK Biobank was 18.3%. Furthermore, this study used the data comprising 17,695 IBS and 129,641 non-IBS (*n* = 147,336) participants to analyze the associations between IBS and its factors. The general characteristics of participants are presented in Table 1. There were 17,695 patients with IBS, and 76.5% of them were women. Over 60% of patients with IBS had high somatization (PHQ-12 score of >6). Sex, age, socioeconomic status, family history of IBS, somatization, antibiotics misuse, coeliac disease, anxiety, and depression were all associated with IBS (*p* <0.001).

**TABLE 1:** Participants’ characteristics.

Variable	All (*n* = 147,336)		*p*-value	Men (*n* = 66,089)		*p*-value	Women (*n* = 81,247)		*p*-value
	IBS *n* = 17,695	Non-IBS *n* = 129,641		IBS *n* = 4,151	Non-IBS *n* = 61,938		IBS *n* = 13,544	Non-IBS *n* = 67,703	
Sex									
Female	13,544 (76.5%)	67,703 (52.2%)	<0.001	NA	NA		NA	NA	
Male	4,151 (23.5%)	61,938 (47.8%)		NA	NA		NA	NA	
Age (years)									
Mean (SD)	54.37 (7.795)	56.14 (7.684)	<0.001	55.02 (7.993)	56.61 (7.760)	<0.001	54.17 (7.722)	55.71 (7.589)	<0.001
Median (IQR)	55 (48, 61)	57 (50, 62)		56 (48, 62)	58 (51, 63)		54 (48.61)	56 (50.62)	
Townsend Deprivation Score									
Mean (SD)	−1.42 (3.007)	−1.73 (2.821)	<0.001	−1.29 (3.156)	−1.78 (2.827)	<0.001	−1.45 (2.959)	−1.69 (2.814)	<0.001
Median (IQR)	−2.24 (−3.70, 0.36)	−2.45 (−3.81, −0.19)		−2.18 (−3.72, 0.57)	−2.51 (−3.85, −0.27)		−2.25 (−3.69, 0.28)	−2.4 (−3.78, −0.12)	
Missing data	30 (0.2%)	149 (0.1%)		10 (0.2%)	67 (0.1%)		20 (0.1%)	82 (0.1%)	
Ever been offered/sought treatment for anxiety									
Yes	6,692 (37.8%)	23,698 (18.3%)	<0.001	1,368 (33.0%)	9,067 (14.6%)	<0.001	5,324 (39.3%)	14,631 (21.6%)	<0.001
No	10,935 (61.8%)	105656 (81.5%)		2,772 (66.8%)	52,759 (85.2%)		8,163 (60.3%)	52,897 (78.1%)	
Missing data	68 (0.4%)	287 (0.2%)		11 (0.3%)	112 (0.2%)		57 (0.4%)	175 (0.3%)	
Ever been offered/sought treatment for depression									
Yes	7,228 (40.8%)	26,498 (20.4%)	<0.001	1,404 (33.8%)	9,849 (15.9%)	<0.001	5,824 (43.0%)	16,649 (24.6%)	<0.001
No	10,391 (58.7%)	102794 (79.3%)		2,732 (65.8%)	51,968 (83.9%)		7,659 (56.5%)	50,826 (75.1%)	
Missing data	76 (0.4%)	349 (0.3%)		15 (0.4%)	121 (0.2%)		61 (0.5%)	228 (0.3%)	
Family history of IBS									
Yes	4,993 (28.2%)	13,985 (10.8%)	<0.001	941 (22.7%)	4,982 (8.0%)	<0.001	4,052 (29.9%)	9,003 (13.3%)	<0.001
No	7,859 (44.4%)	102,225 (78.9%)		1848 (44.5%)	49,500 (79.9%)		6,011 (44.4%)	52,725 (77.9%)	
Missing data	4,843 (27.4%)	13,431 (10.4%)		1,362 (32.8%)	7,456 (12.0%)		3,481 (25.7%)	5,975 (8.8%)	
Born by caesarean section									
Yes	447 (2.5%)	3,298 (2.5%)	0.811	111 (2.7%)	1,598 (2.6%)	0.498	336 (2.5%)	1,700 (2.5%)	0.924
No	16,313 (92.2%)	118899 (91.7%)		3,552 (85.6%)	54,707 (88.3%)		12,761 (94.2%)	64,192 (94.8%)	
Missing data	935 (5.3%)	7,444 (5.7%)		488 (11.8%)	5,633 (9.1%)		447 (3.3%)	1,811 (2.7%)	
Long-term/recurrent antibiotics as child or teenager									
Yes	4,163 (23.5%)	13,997 (10.8%)	<0.001	715 (17.2%)	4,906 (7.9%)	<0.001	3,448 (25.5%)	9,091 (13.4%)	<0.001
No	11,003 (62.2%)	105232 (81.2%)		2,771 (66.8%)	52,078 (84.1%)		8,232 (60.8%)	53,154 (78.5%)	
Missing data	2,529 (14.3%)	10,412 (8.0%)		665 (16.0%)	4,954 (8.0%)		1,864 (13.8%)	5,458 (8.1%)	
Diagnosed with coeliac disease or gluten sensitivity									
Yes	890 (5.0%)	1,462 (1.1%)	<0.001	146 (3.5%)	510 (0.8%)	<0.001	744 (5.5%)	952 (1.4%)	<0.001
No	16,351 (92.4%)	127,701 (98.5%)		3,827 (92.2%)	61,139 (98.7%)		12,524 (92.5%)	66,562 (98.3%)	
Missing data	454 (2.6%)	478 (0.4%)		178 (4.3%)	289 (0.5%)		276 (2.0%)	189 (0.3%)	
PHQ-12 Score									
≤6	5,748 (32.5%)	98,940 (76.3%)	<0.001	1,698 (40.9%)	50,667 (81.8%)	<0.001	8,918 (65.8%)	17,636 (26.0%)	<0.001
>6	11,174 (63.1%)	27,668 (21.3%)		2,256 (54.3%)	10,032 (16.2%)		4,050 (29.9%)	48,273 (71.3%)	
Missing data	773 (4.4%)	3,033 (2.3%)		197 (4.7%)	1,239 (2.0%)		576 (4.3%)	1794 (2.6%)	

Data were mean (SD) or *n* (%) unless noted otherwise. The distribution of age and the Townsend Deprivation Score is non-normal; therefore, the mean (SD) and median (P25, P75) are used to describe. The *p*-value was calculated by the chi-square test and Wilcoxon’s rank-sum test where applicable. In this analysis, “Do not know,” “Prefer not to answer,” and “missing” were coded as missing data.

**FIGURE 2:**
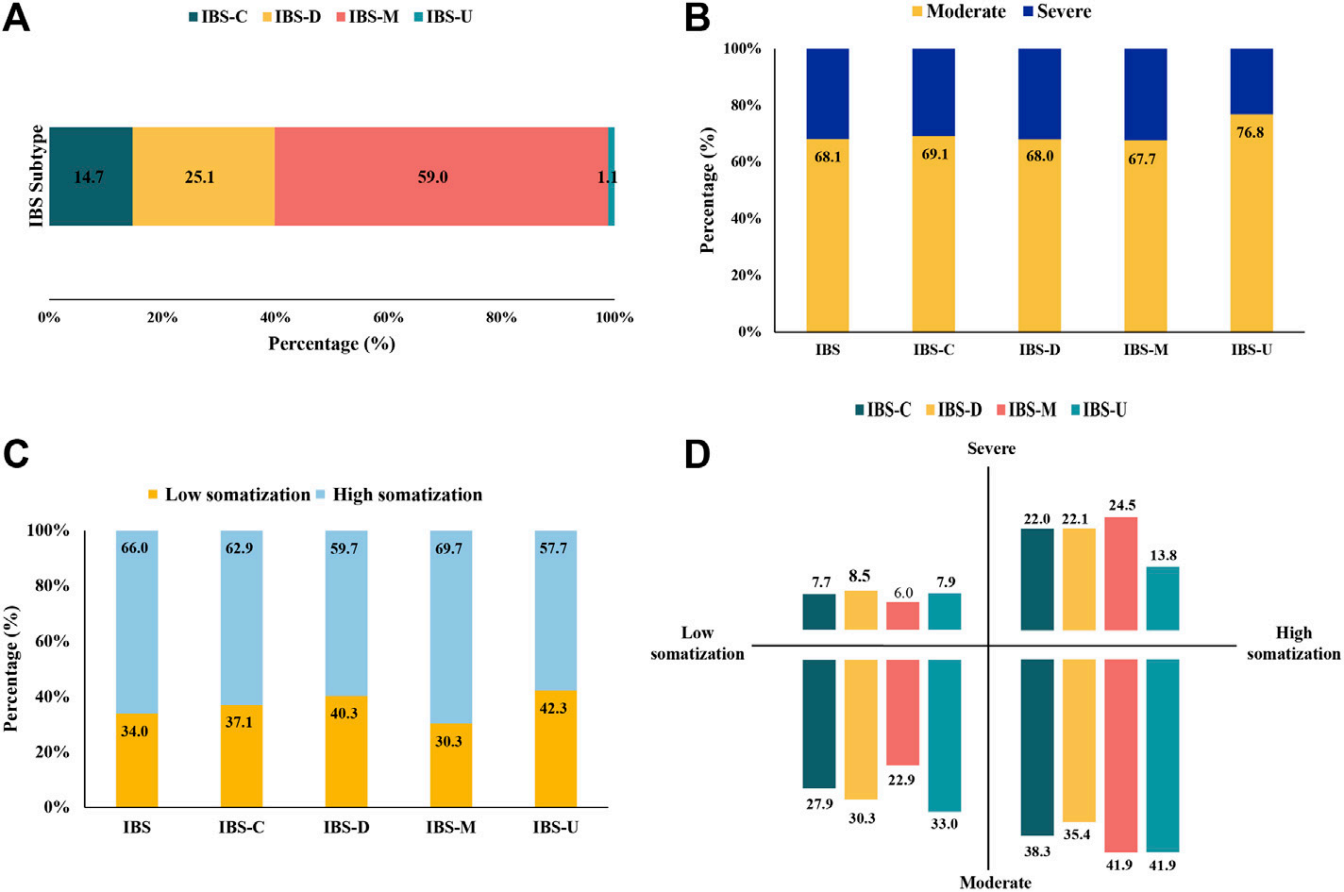
Distribution of IBS subtypes and somatization symptoms. (*n* = 17,695). **(A)** Proportions of participants defined as IBS subtypes. **(B)** Proportions of IBS and its subtypes with moderate and severe symptoms. **(C)** Proportions of IBS and its subtypes with somatization. **(D)** Distribution of IBS subtypes in the field of somatization and symptom severity.

As illustrated in Figure 2 and Supplementary Table S1, IBS-M was predominant, accounting for 59.0% of the cases, followed by IBS-D (25.1%), IBS-C (14.7%), and IBS-U (1.1%). Over 30% of patients with IBS had severe symptoms, including patients with IBS-M (32.3%) having the most and those with IBS-U (23.2%) having the least symptoms. Patients with IBS-M (69.7%) had high somatization, higher than the overall level of IBS (66.0%). IBS-M and IBS-U patients had moderate symptoms and the highest somatization (41.9%).

**FIGURE 3:**
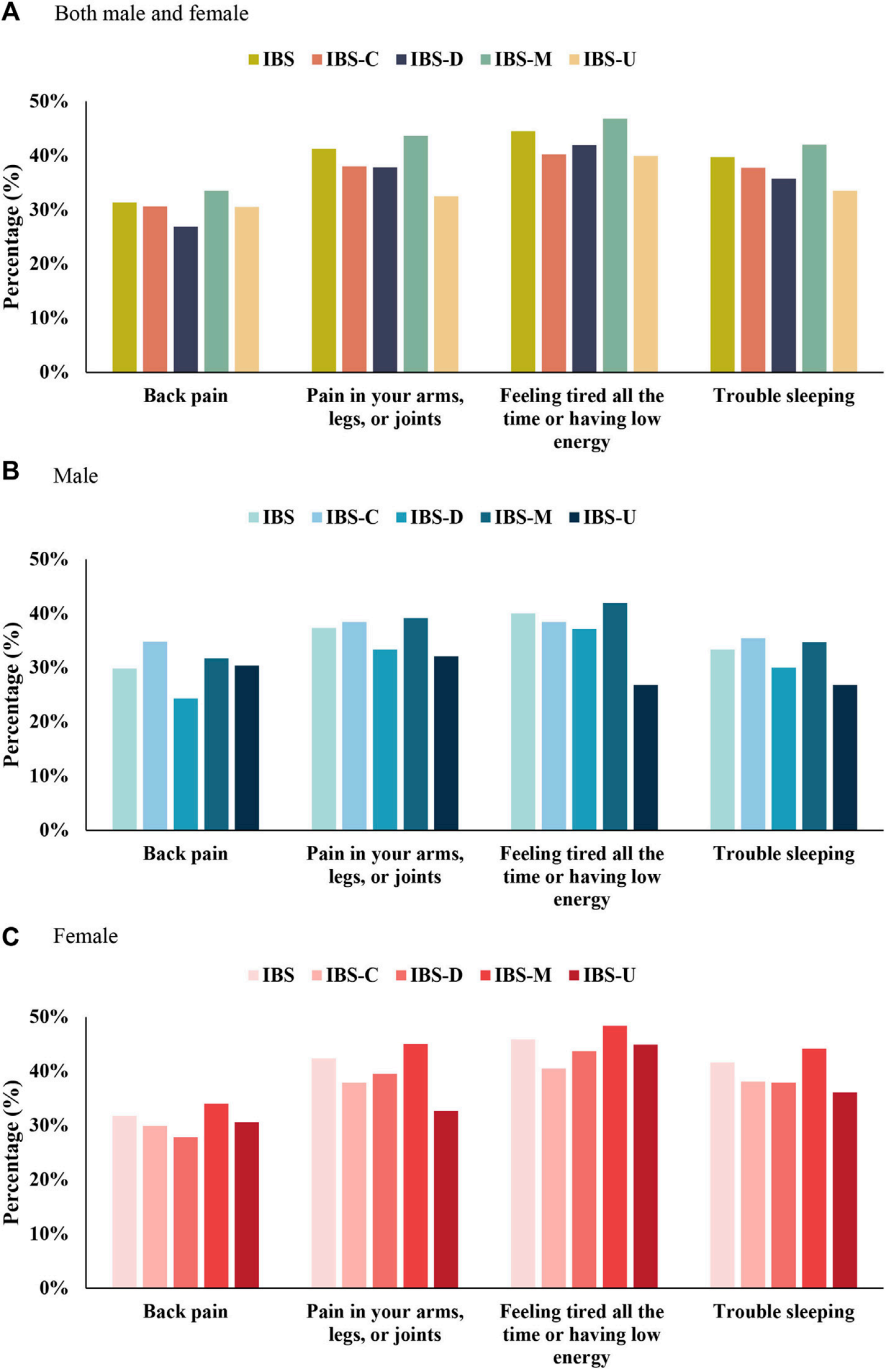
Top four extraintestinal somatic symptoms in IBS and its subtypes. **(A)** Both male and female. **(B)** Male. **(C)** Female.

“Continuously feeling tired or having low energy;” “pain in the arms, legs, or joints;” “trouble sleeping;” and “back pain” were the four major somatic symptoms. Sex-specific differences and subtypes of other somatic symptoms are summarized in Figure 3 and Supplementary Tables S2–S4. A majority of women with IBS suffered from these four major somatic symptoms than men with IBS. A majority of men with IBS-C suffered from back pain and had trouble sleeping than men with IBS-M. Men with IBS-U suffered from low energy and had relatively low sleep problems. In women with IBS-C and IBS-U, having trouble sleeping was more severe than pain in the arms, legs, or joints.

### 3.2 Logistic regression analysis of factors associated with irritable bowel syndrome, stratified by sex

**FIGURE 4:**
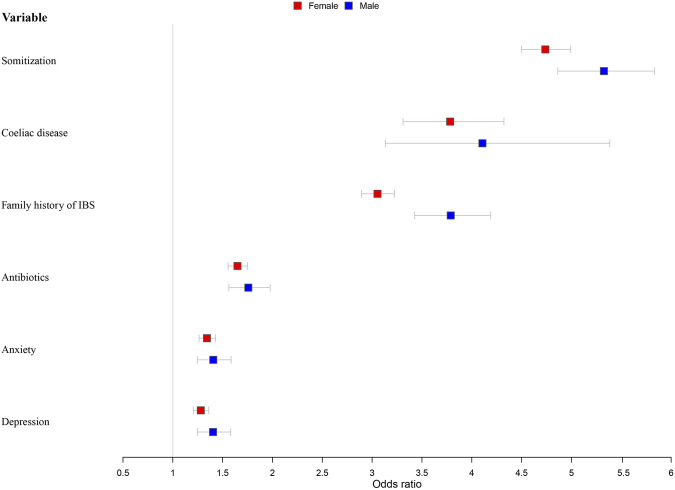
Models of factors associated with IBS stratified by sex and adjusted for age and socioeconomic status.

Independent variables that were significant predictors of IBS in the chi-squared tests or Wilcoxon’s rank-sum test were entered into the logistic regression analysis model (Figure 4; Supplementary Table S5). After adjustment for age and the Townsend Score (Model 2), younger participants were more likely to develop IBS for both men and women. Men with a lower economic level were more likely to have IBS (OR = 1.028, 95% CI = 1.013–1.044). High somatization was the most important influencing factor; people experiencing this symptom were four times more likely to have IBS (male: odds ratio [OR] = 4.786, 95% confidence interval [CI] = 4.544–5.041; female: OR = 5.326, 95% CI = 4.863–5.832). Coeliac disease (male: OR = 4.107, 95% CI = 3.132–5.385; female: OR = 3.783, 95% CI = 3.310–4.323) and family history of IBS (male: OR = 3.789, 95% CI = 3.427–4.190; female: OR = 3.054, 95% CI = 2.893–3.224) were the second important influencing factors; participants with these were about three to four times more susceptible to IBS. Other significant factors included antibiotics abuse (male: OR = 1.758, 95% CI = 1.563–1.977; female: OR = 1.649, 95% CI = 1.555–1.749), anxiety (male: OR = 1.406, 95% CI = 1.247–1.585; female: OR = 1.343, 95% CI = 1.263–1.429), and depression (male: OR = 1.404, 95% CI = 1.248–1.581; female: OR = 1.281, 95% CI = 1.206–1.361).

### 3.3 Multinominal logistic regression analysis of potential influencing factors of different irritable bowel syndrome subtypes, stratified by sex

**FIGURE 5:**
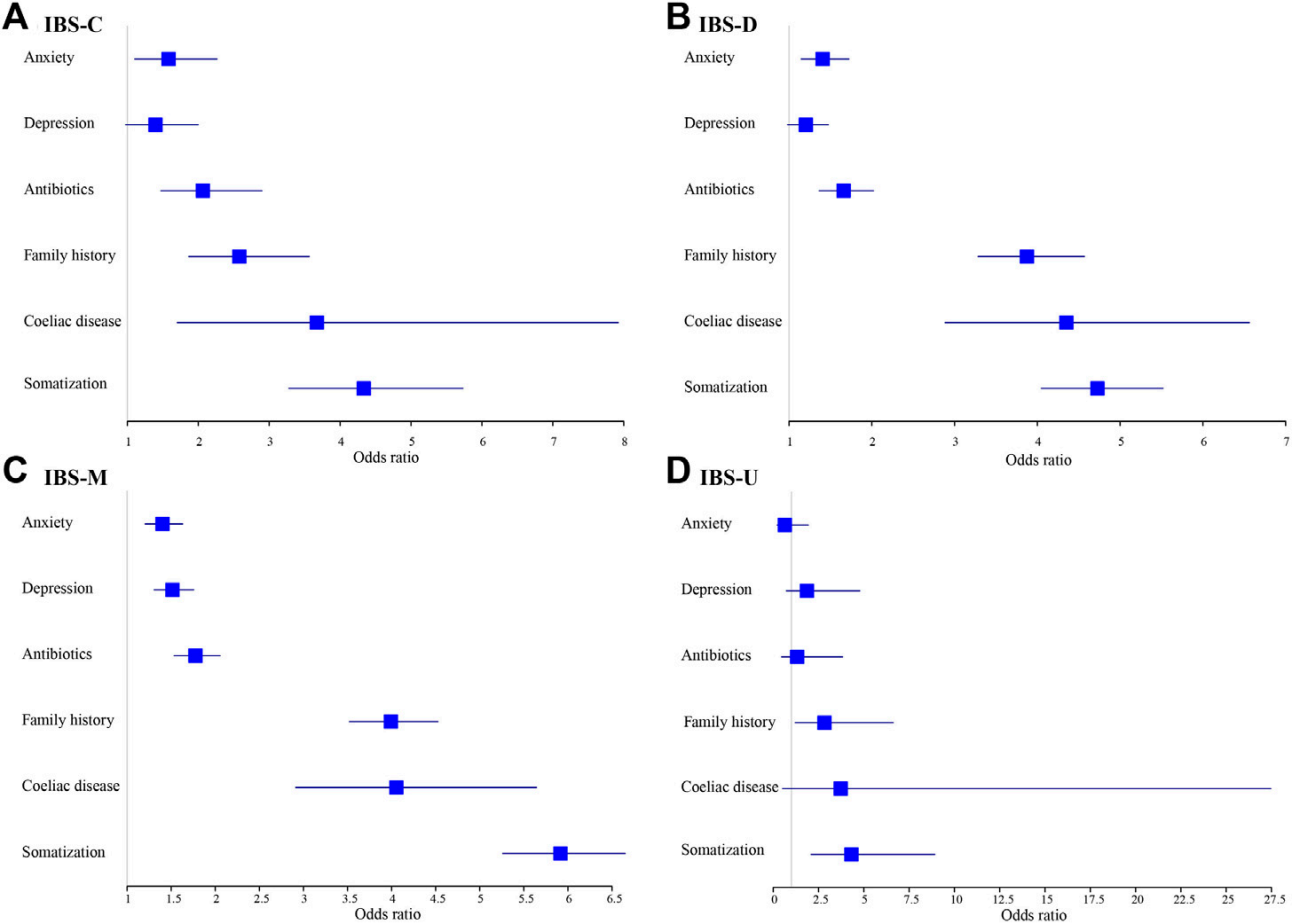
Models of factors associated with IBS subtypes for males and adjusted for age and socioeconomic status. **(A)** IBS-C. **(B)** IBS-D. **(C)** IBS-M. **(D)** IBS-U.

**FIGURE 6:**
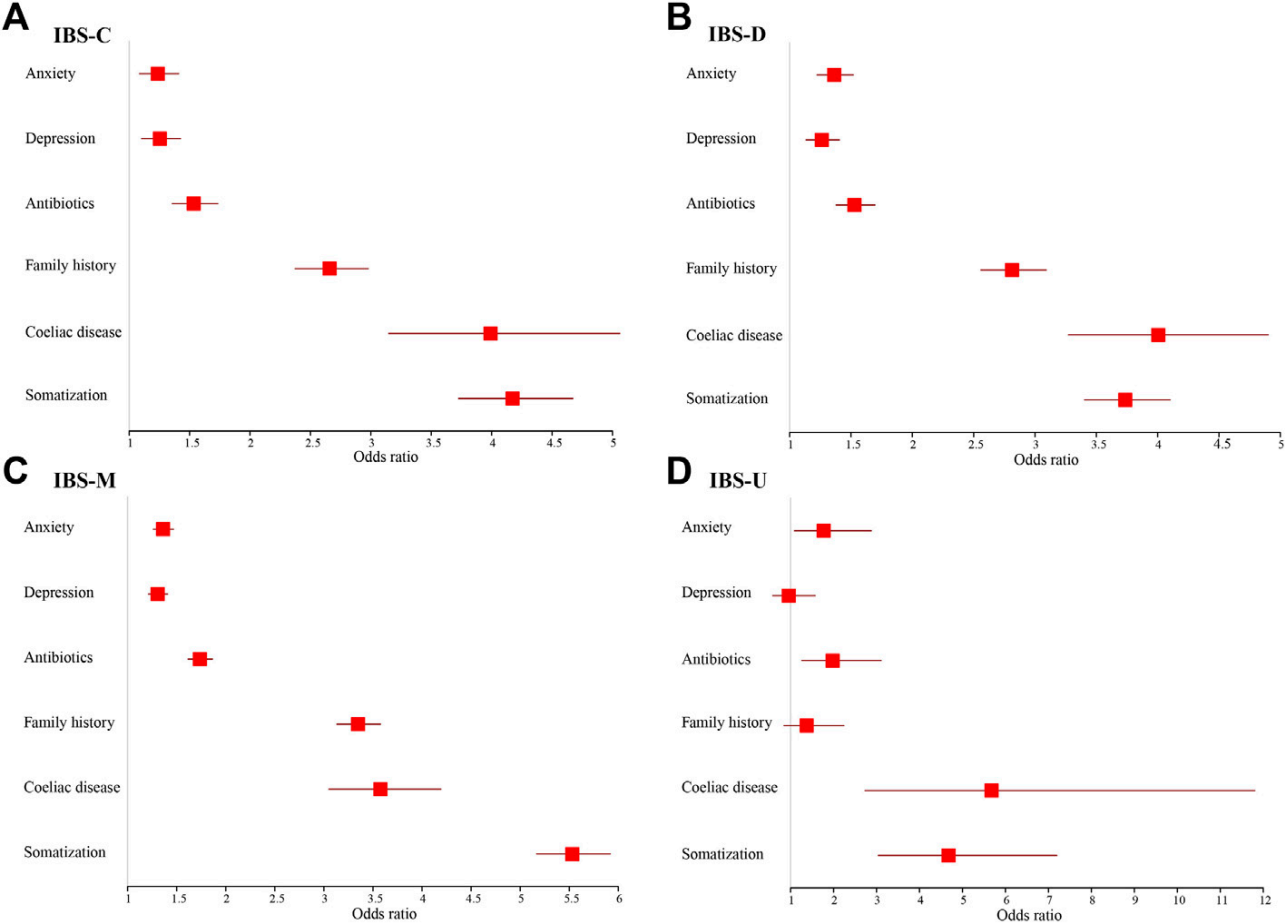
Models of factors associated with IBS subtypes for females and adjusted for age and socioeconomic status. **(A)** IBS-C. **(B)** IBS-D. **(C)** IBS-M. **(D)** IBS-U.

Figures 5, 6 show adjusted OR values (Model 4) of multiple IBS influencing factors for each IBS subtype in men and women (see details in Supplementary Table S6). Somatization and coeliac disease were the top two influencing factors of each IBS subtype for both men and women. Men with high somatization and coeliac disease were most likely to develop IBS-M and IBS-D, up to 5.915 and 4.351 times than men with low somatization and without coeliac disease, respectively. In addition, coeliac disease, antibiotics, and anxiety significantly affected patients with IBS-C, IBS-D, and IBS-M, whereas depression affected only patients with IBS-M (*p* < 0.05).

In women, high somatization ranked first in patients with IBS-C, IBS-M, and IBS-U cases, but coeliac disease ranked first in IBS-D. Women with high somatization suffered up to 5.531, 4.676, and 4.173 times more from IBS-M, IBS-U, and IBS-C, respectively, than women with low somatization. Women with coeliac disease were 4.005 times more likely to develop IBS-D compared to women with high somatization. In addition, women on antibiotics and those with anxiety and depression were significantly more prone to suffer from each IBS subtype (*p* < 0.05), except for women with depression to IBS-U.

### 3.4 Chi-squared analysis of differences in influencing factors among irritable bowel syndrome subtypes

As shown in Table 2, somatization, antibiotics, anxiety, and depression significantly differed among the four IBS subtypes (*p* <0.05). Patients with IBS-M had the highest symptoms, including somatization, anxiety, and depression, with the highest rate being up to 66.4% with high somatization (see Supplementary Tables S7, S8 for sex differences).

**TABLE 2:** Chi-square analysis of differences in influencing factors among different subtypes (*n* = 17,695).

	IBS-C *n* = 2,608	IBS-D *n* = 4,448	IBS-M *n* = 10,436	IBS-U *n* = 203	*p*-value
Sex					<0.001
Female	2,246 (86.1%)	3,239 (72.8%)	7,912 (75.8%)	147 (72.4%)	
Male	362 (13.9%)	1,209 (27.2%)	2,524 (24.2%)	56 (27.6%)	
Age (years)					<0.001
Mean (SD)	54.41 (7.858)	54.26 (7.766)	54.35 (7.788)	57.22 (7.483)	
Median (IQR)	55 (48, 61)	54 (48, 61)	55 (48, 61)	58 (52, 63)	
Townsend Deprivation Score					0.009
Mean (SD)	−1.57 (2.902)	−1.47 (2.944)	−1.35 (3.063)	−1.81 (2.704)	
Median (IQR)	−2.33 (−3.76, 0.01)	−2.26 (−3.71, 0.30)	−2.18 (−3.67, 0.50)	−2.45 (−3.85, −0.53)	
Missing data	4 (0.2%)	7 (0.2%)	17 (0.2%)	2 (1.0%)	
Ever been offered/sought treatment for anxiety					0.003
Yes	931 (35.7%)	1,628 (36.6%)	4,059 (38.9%)	74 (36.5%)	
No	1,667 (63.9%)	2,813 (63.2%)	6,326 (60.6%)	129 (63.5%)	
Missing data	10 (0.4%)	7 (0.2%)	51 (0.5%)	0 (0.0%)	
Ever been offered/sought treatment for depression					<0.001
Yes	1,026 (39.3%)	1713 (38.5%)	4,421 (42.4%)	68 (33.5%)	
No	1,568 (60.1%)	2,721 (61.2%)	5,969 (57.2%)	133 (65.5%)	
Missing data	14 (0.5%)	14 (0.3%)	46 (0.4%)	2 (1.0%)	
Family history of IBS					<0.001
Yes	686 (26.3%)	1,223 (27.5%)	3,048 (29.2%)	36 (17.7%)	
No	1,269 (48.7%)	2082 (46.8%)	4,397 (42.1%)	111 (54.7%)	
Missing data	653 (25.0%)	1,143 (25.7%)	2,991 (28.7%)	56 (27.6%)	
Long-term/recurrent antibiotics as child or teenager					<0.001
Yes	587 (22.5%)	968 (21.8%)	2,562 (24.5%)	46 (22.7%)	
No	1,652 (63.3%)	2,902 (65.2%)	6,316 (60.5%)	133 (65.5%)	
Missing data	369 (14.1%)	578 (13.0%)	1,558 (14.9%)	24 (11.8%)	
Diagnosed with coeliac disease or gluten sensitivity					0.959
Yes	133 (5.1%)	222 (5.0%)	523 (5.0%)	12 (5.9%)	
No	2,408 (92.3%)	4,123 (92.7%)	9,630 (92.3%)	190 (93.6%)	
Missing data	67 (2.6%)	103 (2.3%)	283 (2.7%)	1 (0.5%)	
PHQ-12 Score					<0.001
≤6	928 (35.6%)	1,725 (38.8%)	3,012 (28.9%)	83 (40.9%)	
>6	1,573 (60.3%)	2,555 (57.4%)	6,933 (66.4%)	113 (55.7%)	
Missing data	107 (4.1%)	168 (3.8%)	491 (4.7%)	7 (3.4%)	

Data were mean (SD) or *n* (%) unless noted otherwise. The distribution of age and the Townsend Deprivation Score is non-normal; therefore, the mean (SD) and median (P25 and P75) are used to describe. The *p*-value was calculated by the chi-square test and Wilcoxon’s rank-sum test where applicable. In this analysis, “Do not know,” “Prefer not to answer,” and “missing” were coded as missing data.

### 3.5 Prevalence and influencing factors of post-infectious irritable bowel syndrome

**FIGURE 7:**
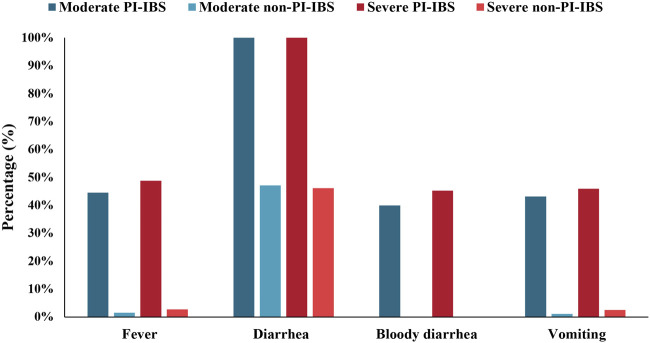
Initial symptoms of participants with PI-IBS and non-PI-IBS stratified by moderate and severe symptoms.

A total of 1,787 individuals met our definition of PI-IBS, and the proportion of PI-IBS in IBS was 1,787/10,760 (16.6%). To further analyze the differences between PI-IBS and non-PI-IBS, eligible participants, including 1,441 PI-IBS and 6,815 non-PI-IBS, were included (*n* = 8,256). A univariate analysis (Table 3) showed statistical differences in anxiety, depression, family history of IBS, antibiotics abuse, coeliac disease, and somatization between PI-IBS and non-PI-IBS groups (*p* <0.05). Figure 7 shows the severity of the initial symptoms in both PI-IBS and non-PI-IBS groups. This study found that all the patients with PI-IBS had diarrhea, whereas almost all non-PI-IBS patients had no bloody diarrhea, vomiting, and fever. In the PI-IBS group, the symptoms of fever, bloody diarrhea, and vomiting were more severe than those in the non-PI-IBS group.

**TABLE 3:** Differences between PI-IBS and non-PI-IBS.

Variable	All (*n* = 8,256)		*p*-value
	PI-IBS *n* = 1,441 (%)	Non-PI-IBS *n* = 6,815 (%)	
Sex			0.011
Female	1,117 (77.5)	5,484 (80.5)	
Male	324 (22.5)	1,331 (19.5)	
Age (years)			<0.001
Mean (SD)	53.27 (7.614)	54.76 (7.722)	
Median (IQR)	53 (47, 59.5)	55 (49, 61)	
Townsend Deprivation Score			<0.001
Mean (SD)	−0.98 (3.168)	−1.56 (2.918)	
Median (IQR)	−1.76 (−3.48, 0.96)	−2.35 (−3.73, 0.13)	
Missing data	2 (0.1)	11 (0.2)	
Ever been offered/sought treatment for anxiety			<0.001
Yes	698 (48.4)	2,916 (42.8)	
No	741 (51.4)	3,867 (56.7)	
Missing data	2 (0.1)	32 (0.5)	
Ever been offered/sought treatment for depression			<0.001
Yes	726 (50.4)	3,017 (44.3)	
No	711 (49.3)	3,767 (55.3)	
Missing data	4 (0.3)	31 (0.5)	
Family history of IBS			0.002
Yes	574 (39.8)	2,382 (35.0)	
No	509 (35.3)	2,629 (38.6)	
Missing data	358 (24.8)	1,804 (26.5)	
Long-term/recurrent antibiotics as child or teenager			<0.001
Yes	496 (34.4)	1,619 (23.8)	
No	712 (49.4)	4,162 (61.1)	
Missing data	233 (16.2)	1,034 (15.2)	
Diagnosed with coeliac disease or gluten sensitivity			<0.001
Yes	139 (9.6)	395 (5.8)	
No	1,246 (86.5)	6,264 (91.9)	
Missing data	56 (3.9)	156 (2.3)	
PHQ-12 Score			<0.001
≤6	271 (18.8)	2,043 (30.0)	
>6	1,097 (76.1)	4,433 (65.0)	
Missing data	73 (5.1)	339 (5.0)	

Data were mean (SD) or *n* (%) unless noted otherwise. The distribution of age and the Townsend Deprivation Score is non-normal; therefore, the mean (SD) and median (P25 and P75) are used to describe. The *p*-value was calculated by the chi-square test and Wilcoxon’s rank-sum test where applicable. In this analysis, “Do not know,” “Prefer not to answer,” and “missing” were coded as missing data.

## 4 Discussion

To the best of our knowledge, this is the first study to estimate risk factors for IBS and its subtypes using the UK Biobank database. The prevalence of IBS reported in this study was relatively high at 18.3%, which was similar to the estimate found in western countries (3%–22%) (Lovell and Ford, 2012). Somatization and coeliac disease were the top two prominent potential influencing factors associated with IBS for both men and women. Meanwhile, the differences between both subtypes and sexes were mainly focused on psychological factors, especially depression. The proportion of PI-IBS in the UK Biobank was 16.6%, and patients with PI-IBS were afflicted by diarrhea compared with non-PI-IBS patients.

The prevalence of IBS in the UK Biobank was 18.3%, which was rational and acceptable. According to the published literatures, the prevalence of IBS in the UK Biobank was still within the scope of UK prevalence ranging from 6.1% to 21.6% (Kennedy and Jones, 2000). Possible reasons for the relatively high prevalence in the UK Biobank were as follows: 1) the UK Biobank attracted healthier volunteers (Fry et al., 2017); however, DHQ decreased “the healthy effect” and amplified the prevalence of IBS in this study. Moreover, 52.1% of participants in the UK Biobank fully completed DHQ after the initial email invitation. Therefore, we considered that DHQ attracted those who were more concerned about digestive health or confused about digestive diseases. 2) Different diagnosis criteria, questionnaires, and methods of questionnaire administration may give rise to differences in the prevalence of IBS (Oka et al., 2020; Sperber et al., 2021). 3) When estimating the prevalence of IBS in the UK Biobank, this study did not restrict a pain/discomfort frequency of at least 2 days a week, similar to that in pathophysiology research and clinical trials (Guthrie et al., 2003). The study followed the general principles to put 2 or 3 days a month, 1 day a week, or more than 1 day a week and comfort or pain on each day as one of the criteria to diagnose IBS.

Somatization was a high-risk factor and ranked first in IBS for both males and females. It reflected patient’s sensitivity to pain and other non-gastrointestinal physical stimuli. This study found that more than 63% of patients with IBS had high somatization, especially for the IBS-M subtype (66.4%), with the feeling of exhaustion all the time or having low energy as the most disturbing somatic symptom (Patel et al., 2015). Somatization was considered a criterion for the diagnosis and treatment of IBS for the instructive understanding of its mechanism. High somatization was caused by visceral hypersensitivity, whereas low somatization was caused by gastrointestinal symptoms (Whitehead et al., 2002; Camilleri and Ford, 2017). Also, high somatization in IBS-M, compared with IBS-C and IBS-D, may be partly explained by more frequency of bloating or abdominal distension and increased levels of anxiety and depression (Patel et al., 2015). This study also found that IBS with severe symptoms had a higher level of somatization than IBS with moderate symptoms (73.4% vs. 58.3%). Somatic symptoms can predict the severity of IBS symptoms, although the tools used were Diagnostic Criteria for Psychosomatic Research-Revised and Somatic Symptom Disorder (PHQ-12 and 7-item Whiteley Index) (Schneider et al., 2017). Therefore, we recommend that doctors should be aware of the connection between somatization and IBS during diagnosis, particularly in patients with IBS-M. Antispasmodics are the most commonly prescribed drugs for IBS and help relieve symptoms of abdominal pain and colic (Volta et al., 2016). However, it is not advisable to blindly use such drugs. Instead, direct treatment of IBS is the more effective method. For example, providing problem-oriented and patient-centered self-management guidelines formulated according to the needs of patients improved the symptoms and patients’ quality of life (Atkinson et al., 2004).

Coeliac disease was another high-risk factor and ranked second in IBS-C, IBS-D, and IBS-M, except in IBS-U in both men and women. Food and diet were the main factors to cause discomfort, and over 80% of IBS respondents reported gastrointestinal symptoms caused by food intolerance (Böhn et al., 2013). For person with coeliac disease, consuming protein gluten found in some whole grains (wheat, rye, and barley) can cause the body’s immune system to attack the small intestine and trigger IBS symptoms (Simrén et al., 2001; Faresjö et al., 2010). Other “trigger foods” of IBS, such as fatty foods, dairy products, coffee, and alcohol, can exacerbate gastrointestinal reactions and increase discomfort; therefore, patients with IBS tend to exhibit visceral hypersensitivity after eating (Hammer et al., 2008; Irvine et al., 2017). For example, capsaicin can provoke visceral pain and hypersensitivity in patients (McKenzie et al., 2016). We observed that 5.0% of patients with IBS had coeliac disease higher than the previous study with 3.3% (National Institute for Health and Care Excellence, 2018). Interestingly, we found that patients with IBS had a higher risk of coeliac disease, with an OR of approximately 3–5 compared with that in non-IBS patients, although there were no differences regarding this factor among IBS subtypes. We may conclude that clinicians can evaluate the possibility of suffering from IBS through coeliac disease, but this factor cannot assist in diagnosing IBS subtypes.

Clinically, doctors are likely to start with a complete disease history, physical exam, and some tests to rule out other conditions in the absence of effective inspection tools. However, an excessive inspection may take place. In view of the close relationship with diet, doctors should carefully inquire about the patient’s dietary habits and discomfort of the digestive system for the diagnosis to provide individualized treatment in accordance with the appropriate IBS subtypes, such as IBS-U and IBS-D for women and IBS-M and IBS-D for men. For the treatment of IBS, clinicians may consider recommending that patients undertake dietary therapy based on guidance provided by the National Institute for Health and Care Excellence and the British Dietetic Association (Maxwell et al., 2002; Staudacher et al., 2012). In addition, a low or free fermentable oligosaccharides, disaccharides, monosaccharides, and polyols (FODMAPs) diet was an effective therapy (Pimentel et al., 2011); however, clinicians should emphasize a balanced diet instead of a single diet.

Long-term/recurrent use of antibiotics in children or adolescents was a relatively new variable to assess the influencing factors for IBS and its subtypes, except for IBS-U. We found that 12.3% of patients had a history of antibiotic exposure, and IBS-M was the most affected, which aligns with the results of a previous study (Krogsgaard et al., 2018). Antibiotics could affect the gut microbiota, which plays an important role in the occurrence and development of IBS. First, consistent alterations in the gut microbiota of patients with IBS may remind a significant association between gut microbiota and IBS pathophysiology (Sharpe et al., 1992). Second, the gut microbiota may involve and alter the construction of the gut epithelial barrier (Kennedy et al., 2014). Third, the gut microbiota is involved in the brain–gut axis and has effects on the pathogenesis of depression and anxiety (Drossman and Hasler, 2016). Antibiotics can modulate anxious and depressive behavior by modulating the gut microbiota (Tait and Sayuk, 2021). However, antibiotics not only modulated the gut microbiota but also disrupted the normal gut microbiota. They also triggered the alterations of the gut microbiota and gastrointestinal motility and led to chronic gastrointestinal symptoms (Thiwan and Drossman, 2006; Kinsinger, 2017). Therefore, antibiotics may not only exacerbate but also improve the symptoms of IBS (Klem et al., 2017; Berumen et al., 2020). To conclude, the use of antibiotics may be considered a “double-edged sword”, and problem-oriented and patient-centered guidelines should be formulated. Clinicians could use antibiotics for short-term therapeutic effects, although they need to be carefully considered for long-term use. To some extent, this study has confirmed that antibiotic abuse in children was a potential risk factor for IBS. However, the inherent link between the long-term use of antibiotics and the mechanism of IBS remains unproven. Therefore, greater emphasis and attention should be placed on proving the impacts of different kinds and duration of use of antibiotics on IBS and its subtypes.

Mental disorders were recognized risk factors for IBS. Patients with IBS exhibited higher anxious and depressive tendencies (Cremonini and Talley, 2005), and about 40% of women with IBS had mental problems in this study. Furthermore, over 20% of people with mental disabilities had IBS, which may indicate a potential interaction between psychological factors and IBS. The brain–gut axis, the bidirectional communication mechanism between the gut and the central nervous system, is usually used to explain this phenomenon (Corsetti and Linaclotide, 2013; Canavan et al., 2014b). Pain perception, emotional arousal, and cognitive response formed by the brain-affected bowel movement and secretion provided a top-down communication conduction method. In contrast, intestinal function regulated the central nervous system and promoted intestinal responses to emotions and cognition, which provided bottom-up communication (Porcelli et al., 2020). Based on the model of IBS pathogenesis, non-pharmacological treatments have also been proposed; commonly used psychological therapies are cognitive behavioral therapy and hypnotherapy (Duan et al., 2019). It is important that antidepressants are considered as treatment options for IBS and are prioritized or recommended to patients with both IBS and depression as a safe, long-term drug treatment method (Piche et al., 2009).

In this study, the prevalence of PI-IBS in the UK Biobank was 16.6% among patients with IBS, which was a logical reference to other literature (Schwille-Kiuntke et al., 2011; Johnsen et al., 2018). Some patients with PI-IBS (about 10%) eventually develop IBS (Heenan et al., 2020). This may indicate that PI-IBS does not have long-term stability and ultimately transits to IBS. This study found that patients with PI-IBS suffered from diarrhea, although this could not determine the differences between PI-IBS and non-PI-IBS. Instead, bloody diarrhea may be considered in the diagnosis of PI-IBS. Once diagnosed with PI-IBS, the patient’s prognosis is likely to improve rather than worsen (Bercik et al., 2011); therefore, attention should be paid to the severity of symptoms in patients with PI-IBS. This study believed that more attention should be paid to the effects of antibiotics on patients with PI-IBS. A previous study reported that the patients who were given antibiotics following infection were more likely to develop longer-lasting IBS symptoms (Mearin et al., 2005; Spiller and Garsed, 2009; Ghoshal et al., 2012; Ringel and Maharshak, 2013).

This study used high-quality large sample data with 174,217 participants from the UK Biobank to explore and analyze the differences in influencing factors of IBS in terms of sexes and subtypes. This study found that the pooled prevalence of IBS in the UK Biobank was approximately 18.3%, of which the prevalence of PI-IBS was 16.6%. This study reveals that somatization and coeliac disease are the most prominent influencing factors of IBS and its subtypes. Considerable differences associated with sex and subtypes are reflected in psychological factors and coeliac disease. The main results of this study were to comprehensively demonstrate the differences of influencing factors based on sex and subtypes. This research could provide guidance for clinicians and suggest that special attention is required regarding sex-specific differences. Moreover, this study emphasizes the importance of psychological treatment and adoption of particular treatment according to each subtype. However, there were several limitations in the UK Biobank database that should be considered. First, this study was a cross-sectional study using the UK Biobank database. Therefore, this study could not establish causal relationships based on the results. Second, the UK Biobank may attract participants who are more concerned with digestive health and their self-reported exposures and outcomes may cause reporting bias. This study may not represent the full picture of the UK; however, we still put emphasis on the advantages and use of the UK Biobank database because this study aimed to explore the differences of factors associated with IBS subtypes rather than the epidemiological features of IBS. Third, the DHQ was designed by a group of experts in 2015, when Rome III was still the common standard for diagnosing IBS. Sadly, at the time of the 2017 study, the questionnaire had not been updated, which posed some limitations to the study. Future studies should use the latest IBS diagnostic tools to explore and compare the relationships between various influencing factors and to identify the mediators and moderators that affect IBS. Moreover, longitudinal research exploring the causal relationship between various risk factors and IBS is required.

## Data Availability

The datasets presented in this study can be found in online repositories. The names of the repository/repositories and accession number(s) can be found at: https://www.ukbiobank.ac.uk/; application number 56320.
