# Supplementary material for: Factors related to irritable bowel syndrome and differences among subtypes: A cross-sectional study in the UK Biobank
Source: Front Pharmacol. 2022 Aug 26;13:905564. doi: 10.3389/fphar.2022.905564 (PMC9458926; doi:10.3389/fphar.2022.905564)
Supplement: Supplementary file 1 [file DataSheet1.docx]

Supplementary Material

# Supplementary Data

Table S1. The relationship between PHQ score and IBS severity

| **Variable** | **Total IBS subtypes (n = 17695)** | | | | **p value** | **Moderate (n = 12052)** | | | | **p value** | **Severe (n = 5643)** | | | | **p value** |
| --- | --- | --- | --- | --- | --- | --- | --- | --- | --- | --- | --- | --- | --- | --- | --- |
|  | **IBS-C n=2608**  **(%)** | **IBS-D n=4448**  **(%)** | **IBS-M n=10436**  **(%)** | **IBS-U n=203**  **(%)** |  | **IBS-C n=1803**  **(%)** | **IBS-D n=3024 (%)** | **IBS-M n=7069**  **(%)** | **IBS-U n=156 (%)** |  | **IBS-C n=805**  **(%)** | **IBS-D n=1424**  **(%)** | **IBS-M n=3367**  **(%)** | **IBS-U n=47**  **(%)** |  |
| Low somatisation | 928  (35.6) | 1725  (38.8) | 3012  (28.9) | 83  (40.9) | <0.001 | 728  (40.4) | 1349  (44.6) | 2390  (33.8) | 67  (42.9) | <0.001 | 200  (24.8) | 376  (26.4) | 622  (18.5) | 16  (34.0) | <0.001 |
| High somatisation | 1573  (60.3) | 2555  (57.4) | 6933  (66.4) | 113  (55.7) |  | 999  (55.4) | 1573  (52.0) | 4374  (61.9) | 85  (54.5) |  | 574  (71.3) | 982  (69.0) | 2559  (76.0) | 28  (59.6) |  |
| Missing data | 107  (4.1) | 168  (3.8) | 491  (4.7) | 7  (3.4) |  | 76  (4.2) | 102  (3.4) | 305  (4.3) | 4  (2.6) |  | 31  (3.9) | 66  (4.6) | 186  (5.5) | 3  (6.4) |  |

Table S2. Chi-square analysis of the relationship between extraintestinal somatic symptoms and IBS and subtypes (Total)

|  | | **Total IBS**  **n = 17695(%)** | | **IBS subtypes** | | | | | | | | | | | | |  | **p value** |
| --- | --- | --- | --- | --- | --- | --- | --- | --- | --- | --- | --- | --- | --- | --- | --- | --- | --- | --- |
|  |  |  |  | **IBS-C** | | **IBS-D** | | | | **IBS-M** | | | **IBS-U** | | | |  |  |
|  |  |  |  | **n = 2608 (%)** | | **n = 4448 (%)** | | | | **n = 10436 (%)** | | | **n = 203(%)** | | | |  |  |
| **Back pain** | |  | |  |  |  | |  | |  |  | |  | |  | |  | <0.001 |
| Not bothered at all | | 3577 | (20.2) | 568 | (21.8) | 1053 | | (23.7) | | 1904 | (18.2) | | 52 | | (25.6) | |  |  |
| Bothered a little | | 8567 | (48.4) | 1243 | (47.7) | 2199 | | (49.4) | | 5036 | (48.2) | | 89 | | (43.8) | |  |  |
| Bothered a lot | | 5547 | (31.3) | 797 | (30.6) | 1196 | | (26.9) | | 3492 | (33.5) | | 62 | | (30.5) | |  |  |
| Missing data | | 4 | (＜0.1) | 0 | (0) | 0 | | (0) | | 4 | (＜0.1) | | 0 | | (0) | |  |  |
| **Pain in your arms, legs, or joints** | | | | | | | |  | |  |  | |  | |  | |  | <0.001 |
| Not bothered at all | | 2568 | (14.5) | 435 | (16.7) | 749 | | (16.8) | | 1344 | (12.9) | | 40 | | (19.7) | |  |  |
| Bothered a little | | 7834 | (44.3) | 1182 | (45.3) | 2018 | | (45.4) | | 4537 | (43.5) | | 97 | | (47.8) | |  |  |
| Bothered a lot | | 7286 | (41.2) | 991 | (38.0) | 1681 | | (37.8) | | 4548 | (43.6) | | 66 | | (32.5) | |  |  |
| Missing data | | 7 | (＜0.1) | 0 | (0) | 0 | | (0) | | 7 | (＜0.1) | | 0 | | (0) | |  |  |
| **Headaches** | | | | |  |  | |  | |  |  | |  | |  | |  | <0.001 |
| Not bothered at all | | 6714 | (37.9) | 982 | (37.7) | 1957 | | (44.0) | | 3679 | (35.2) | | 96 | | (47.3) | |  |  |
| Bothered a little | | 8646 | (48.9) | 1283 | (49.2) | 1995 | | (44.9) | | 5282 | (50.6) | | 86 | | (42.3) | |  |  |
| Bothered a lot | | 2330 | (13.2) | 343 | (13.2) | 494 | | (11.1) | | 1472 | (14.1) | | 21 | | (10.3) | |  |  |
| Missing data | | 5 | (＜0.1) | 0 | (0) | 2 | | (＜0.1) | | 3 | (＜0.1) | | 0 | | (0) | |  |  |
| **Chest pain** | | | | |  |  | |  | |  |  | |  | |  | |  | <0.001 |
| Not bothered at all | | 12403 | (70.1) | 1897 | (72.7) | 3319 | | (74.6) | | 7043 | (67.5) | | 144 | | (70.9) | |  |  |
| Bothered a little | | 4589 | (25.9) | 627 | (24.0) | 983 | | (21.1) | | 2925 | (28.0) | | 54 | | (26.6) | |  |  |
| Bothered a lot | | 688 | (3.9) | 82 | (3.1) | 142 | | (3.2) | | 459 | (4.4) | | 5 | | (2.5) | |  |  |
| Missing data | | 15 | (＜0.1) | 2 | (＜0.1) | 4 | | (＜0.1) | | 9 | (＜0.1) | | 0 | | (0) | |  |  |
| **Dizziness** | |  | |  |  |  | |  | |  |  | |  | |  | |  | <0.001 |
| Not bothered at all | | 9245 | (52.2) | 1419 | (54.4) | 2489 | | (56.0) | | 5218 | (50) | | 119 | | (58.6) | |  |  |
| Bothered a little | | 7289 | (41.2) | 1050 | (40.3) | 1696 | | (38.1) | | 4466 | (42.8) | | 77 | | (37.9) | |  |  |
| Bothered a lot | | 1154 | (6.5) | 137 | (5.2) | 260 | | (5.8) | | 750 | (7.2) | | 7 | | (3.4) | |  |  |
| Missing data | | 7 | (＜0.1) | 2 | (＜0.1) | 3 | | (＜0.1) | | 2 | (＜0.1) | | 0 | | (0) | |  |  |
| **Fainting spells** | | | | |  |  | |  | |  |  | |  | |  | |  | 0.019 |
| Not bothered at all | | 16346 | (92.4) | 2425 | (93.0) | 4154 | | (93.4) | | 9581 | (91.8) | | 186 | | (91.6) | |  |  |
| Bothered a little | | 1215 | (6.9) | 163 | (6.3) | 263 | | (5.9) | | 772 | (7.4) | | 17 | | (8.4) | |  |  |
| Bothered a lot | | 108 | (0.6) | 18 | (0.7) | 24 | | (0.5) | | 66 | (0.6) | | 0 | | (0) | |  |  |
| Missing data | | 26 | (0.1) | 2 | (＜0.1) | 7 | | (0.2) | | 17 | (0.2) | | 0 | | (0) | |  |  |
| **Feeling your heart pound or race** | | | | | | | |  | |  |  | |  | |  | |  | <0.001 |
| Not bothered at all | | 8519 | (48.1) | 1325 | (50.8) | 2303 | | (51.8) | | 4776 | (45.8) | | 115 | | (56.7) | |  |  |
| Bothered a little | | 7448 | (42.1) | 1039 | (39.8) | 1750 | | (39.3) | | 4589 | (44.0) | | 70 | | (23.3) | |  |  |
| Bothered a lot | | 1719 | (9.7) | 244 | (9.4) | 394 | | (8.9) | | 1063 | (10.2) | | 18 | | (8.9) | |  |  |
| Missing data | | 9 | (＜0.1) | 0 | (0) | 1 | | (＜0.1) | | 8 | (＜0.1) | | 0 | | (0) | |  |  |
| **Shortness of breath** | | | | | |  | |  | |  |  | |  | |  | |  | <0.001 |
| Not bothered at all | | 9224 | (52.1) | 1498 | (57.4) | 2402 | | (54.0) | | 5215 | (50) | | 109 | | (53.7) | |  |  |
| Bothered a little | | 6633 | (37.5) | 900 | (34.5) | 1618 | | (36.4) | | 4037 | (38.7) | | 78 | | (38.4) | |  |  |
| Bothered a lot | | 1828 | (10.3) | 210 | (8.1) | 422 | | (9.5) | | 1180 | (11.3) | | 16 | | (7.9) | |  |  |
| Missing data | | 10 | (＜0.1) | 0 | (0) | 6 | | (0.1) | | 4 | (＜0.1) | | 0 | | (0) | |  |  |
| **Pain or problems during intercourse** | | | | | | | |  |  | |  |  | |  | |  | | <0.001 |
| Not bothered at all | 6204 | | (35.1) | 867 | (33.2) | | 1758 | (39.5) | 3501 | | (33.5) | 78 | | (38.4) | |  | |  |
| Bothered a little | 2557 | | (14.5) | 389 | (14.9) | | 566 | (12.7) | 1581 | | (15.1) | 21 | | (10.3) | |  | |  |
| Bothered a lot | 1368 | | (7.7) | 240 | (9.2) | | 287 | (6.5) | 831 | | (8.0) | 10 | | (4.9) | |  | |  |
| Missing data | 7566 | | (42.8) | 1112 | (42.6) | | 1837 | (41.3) | 4523 | | (43.3) | 94 | | (46.3) | |  | |  |
| **Feeling tired all the time or having low energy** | | | | | | | | | |  |  | |  | |  | |  | <0.001 |
| Not bothered at all | | 1961 | (11.1) | 341 | (13.1) | 561 | | (12.6) | | 1028 | (9.9) | | 31 | | (15.3) | |  |  |
| Bothered a little | | 7850 | (44.4) | 1219 | (46.7) | 2023 | | (45.5) | | 4517 | (43.3) | | 91 | | (44.8) | |  |  |
| Bothered a lot | | 7878 | (44.5) | 1048 | (40.2) | 1863 | | (41.9) | | 4886 | (46.8) | | 81 | | (39.9) | |  |  |
| Missing data | | 6 | (＜0.1) | 0 | (0) | 1 | | (＜0.1) | | 5 | (＜0.1) | | 0 | | (0) | |  |  |
| **Trouble sleeping** | | | | |  |  | |  | |  |  | |  | |  | |  | <0.001 |
| Not bothered at all | | 2805 | (15.9) | 433 | (16.6) | 820 | | (18.4) | | 1499 | (14.4) | | 53 | | (26.1) | |  |  |
| Bothered a little | | 7863 | (44.4) | 1190 | (45.6) | 2039 | | (45.8) | | 4552 | (43.6) | | 82 | | (40.4) | |  |  |
| Bothered a lot | | 7017 | (39.7) | 983 | (37.7) | 1588 | | (35.7) | | 4378 | (42.0) | | 68 | | (33.5) | |  |  |
| Missing data | | 10 | (＜0.1) | 2 | (＜0.1) | 1 | | (＜0.1) | | 7 | (＜0.1) | | 0 | | (＜0.1) | |  |  |

The question for “menstrual cramps or other problems with the period” was taken into the analysis only in female sex. In this analysis, “Do not know”, “Prefer not to answer” and “missing” were coded as missing data.

Table S3. Chi-square analysis of the relationship between extraintestinal somatic symptoms and IBS and subtypes (Males)

|  | **IBS**  **n = 4151 (%)** | | **IBS subtypes** | | | | | | | |  |  |
| --- | --- | --- | --- | --- | --- | --- | --- | --- | --- | --- | --- | --- |
|  |  |  | **IBS-C** | | **IBS-D** | | **IBS-M** | | **IBS-U** | |  | **p-value** |
|  |  |  | **n = 362 (%)** | | **n = 1209 (%)** | | **n = 2524 (%)** | | **n = 56(%)** | |  |  |
| **Back pain** |  |  |  |  |  |  |  |  |  |  |  | <0.001 |
| Not bothered at all | 943 | (22.7) | 82 | (22.7) | 300 | (24.8) | 550 | (21.8) | 11 | (19.6) |  |  |
| Bothered a little | 1969 | (47.4) | 154 | (42.5) | 615 | (50.9) | 1172 | (46.4) | 28 | (50) |  |  |
| Bothered a lot | 1238 | (29.8) | 126 | (34.8) | 294 | (24.3) | 801 | (31.7) | 17 | (30.4) |  |  |
| Missing data | 1 | (＜0.1) | 0 | (0) | 0 | (0) | 1 | (＜0.1) | 0 | (0) |  |  |
| **Pain in your arms, legs, or joints** | | | | | |  |  |  |  |  |  | 0.026 |
| Not bothered at all | 765 | (18.4) | 69 | (19.1) | 248 | (20.5) | 437 | (17.3) | 11 | (19.6) |  |  |
| Bothered a little | 1837 | (44.3) | 154 | (42.5) | 558 | (46.2) | 1098 | (43.5) | 27 | (48.2) |  |  |
| Bothered a lot | 1548 | (37.3) | 139 | (38.4) | 403 | (33.3) | 988 | (39.1) | 18 | (32.1) |  |  |
| Missing data | 1 | (＜0.1) | 0 | (0) | 0 | (0) | 1 | (＜0.1) | 0 | (0) |  |  |
| **Headaches** | | | |  |  |  |  |  |  |  |  | 0.001 |
| Not bothered at all | 2006 | (48.3) | 178 | (49.2) | 646 | (53.4) | 1151 | (45.6) | 31 | (55.4) |  |  |
| Bothered a little | 1735 | (41.8) | 147 | (40.6) | 454 | (37.6) | 1115 | (44.2) | 19 | (33.9) |  |  |
| Bothered a lot | 410 | (9.9) | 37 | (10.2) | 109 | (9.0) | 258 | (10.2) | 6 | (10.7) |  |  |
| Missing data | 0 | (0) | 0 | (0) | 0 | (0) | 0 | (0) | 0 | (0) |  |  |
| **Chest pain** | | | |  |  |  |  |  |  |  |  | <0.001 |
| Not bothered at all | 2662 | (64.1) | 220 | (60.8) | 850 | (70.3) | 1555 | (61.6) | 37 | (66.1) |  |  |
| Bothered a little | 1269 | (30.6) | 127 | (35.1) | 304 | (25.1) | 821 | (32.5) | 17 | (30.4) |  |  |
| Bothered a lot | 220 | (5.3) | 15 | (4.1) | 55 | (4.5) | 148 | (5.9) | 2 | (3.6) |  |  |
| Missing data | 0 | (0) | 0 | (0) | 0 | (0) | 0 | (0) | 0 | (0) |  |  |
| **Dizziness** |  |  |  |  |  |  |  |  |  |  |  | <0.001 |
| Not bothered at all | 2310 | (55.6) | 208 | (57.5) | 738 | (61.0) | 1327 | (52.6) | 37 | (66.1) |  |  |
| Bothered a little | 1590 | (38.3) | 131 | (36.2) | 410 | (33.9) | 1034 | (41.0) | 15 | (26.8) |  |  |
| Bothered a lot | 251 | (6.0) | 23 | (6.4) | 61 | (5.0) | 163 | (6.5) | 4 | (7.1) |  |  |
| Missing data | 0 | (0) | 0 | (0) | 0 | (0) | 0 | (0) | 0 | (0) |  |  |
| **Fainting spells** | | | |  |  |  |  |  |  |  |  | 0.335 |
| Not bothered at all | 3777 | (91.0) | 335 | (92.5) | 1111 | (91.9) | 2278 | (90.3) | 53 | (94.6) |  |  |
| Bothered a little | 335 | (8.1) | 22 | (6.1) | 88 | (7.3) | 222 | (8.8) | 3 | (5.4) |  |  |
| Bothered a lot | 37 | (0.9) | 5 | (1.4) | 10 | (0.8) | 22 | (0.9) | 0 | (0) |  |  |
| Missing data | 2 | (＜0.1) | 0 | (0) | 0 | (0) | 2 | (＜0.1) | 0 | (0) |  |  |
| **Feeling your heart pound or race** | | | | | |  |  |  |  |  |  | 0.027 |
| Not bothered at all | 2337 | (56.3) | 217 | (59.9) | 719 | (59.5) | 1366 | (54.1) | 35 | (62.5) |  |  |
| Bothered a little | 1486 | (35.8) | 114 | (31.5) | 409 | (33.8) | 945 | (37.4) | 18 | (32.1) |  |  |
| Bothered a lot | 325 | (7.8) | 31 | (8.6) | 81 | (6.7) | 210 | (8.3) | 3 | (5.4) |  |  |
| Missing data | 3 | (＜0.1) | 0 | (0) | 0 | (0) | 3 | (0.1) | 0 | (0) |  |  |
| **Shortness of breath** | | | | |  |  |  |  |  |  |  | 0.033 |
| Not bothered at all | 2036 | (49.0) | 179 | (49.4) | 625 | (51.7) | 1205 | (47.7) | 27 | (48.2) |  |  |
| Bothered a little | 1589 | (38.3) | 135 | (37.3) | 458 | (37.9) | 970 | (38.4) | 26 | (46.4) |  |  |
| Bothered a lot | 523 | (12.6) | 48 | (13.3) | 125 | (10.3) | 347 | (13.7) | 3 | (5.4) |  |  |
| Missing data | 3 | (＜0.1) | 0 | (0) | 1 | (0.1) | 2 | (0.1) | 0 | (0) |  |  |
| **Pain or problems during intercourse** | | | | | |  |  |  |  |  |  | 0.010 |
| Not bothered at all | 2160 | (52.0) | 175 | (48.3) | 679 | (56.2) | 1273 | (50.4) | 33 | (58.9) |  |  |
| Bothered a little | 478 | (11.5) | 28 | (7.7) | 125 | (10.3) | 322 | (12.8) | 3 | (5.4) |  |  |
| Bothered a lot | 171 | (4.1) | 19 | (5.2) | 46 | (3.8) | 105 | (4.2) | 1 | (1.8) |  |  |
| Missing data | 1342 | (32.3) | 140 | (38.7) | 359 | (29.7) | 824 | (32.6) | 19 | (33.9) |  |  |
| **Feeling tired all the time or having low energy** | | | | | | |  |  |  |  |  | 0.002 |
| Not bothered at all | 549 | (13.2) | 49 | (13.5) | 188 | (15.6) | 299 | (11.8) | 13 | (23.2) |  |  |
| Bothered a little | 1940 | (46.7) | 174 | (48.1) | 572 | (47.3) | 1166 | (46.2) | 28 | (50.0) |  |  |
| Bothered a lot | 1661 | (40) | 139 | (38.4) | 449 | (37.1) | 1058 | (41.9) | 15 | (26.8) |  |  |
| Missing data | 1 | (＜0.1) | 0 | (0) | 0 | (0) | 1 | (＜0.1) | 0 | (0) |  |  |
| **Trouble sleeping** | | | |  |  |  |  |  |  |  |  | 0.007 |
| Not bothered at all | 909 | (21.9) | 87 | (24.0) | 290 | (24.0) | 514 | (20.4) | 18 | (32.1) |  |  |
| Bothered a little | 1857 | (44.7) | 146 | (40.3) | 557 | (46.1) | 1131 | (44.8) | 23 | (41.1) |  |  |
| Bothered a lot | 1382 | (33.3) | 128 | (35.4) | 362 | (30) | 877 | (34.7) | 15 | (26.8) |  |  |
| Missing data | 3 | (＜0.1) | 1 | (0.3) | 0 | (0) | 2 | (0.1) | 0 | (0) |  |  |

The question for “menstrual cramps or other problems with the period” was taken into the analysis only in female sex. In this analysis, “Do not know”, “Prefer not to answer” and “missing” were coded as missing data.

Table S4. Chi-square analysis on the relationship between extraintestinal somatic symptoms and IBS and subtypes (Females)

|  | **IBS**  **n = 13544 (%)** | | | | **IBS subtypes** | | | | | | | | |  |  |
| --- | --- | --- | --- | --- | --- | --- | --- | --- | --- | --- | --- | --- | --- | --- | --- |
|  |  |  |  |  | **IBS-C** | | | **IBS-D** | | **IBS-M** | | **IBS-U** | |  | **p-value** |
|  |  |  |  |  | **n = 2246 (%)** | | | **n = 3239 (%)** | | **n = 7912 (%)** | | **n = 147 (%)** | |  |  |
| **Back pain** |  | |  | |  | |  |  |  |  |  |  |  |  | <0.001 |
| Not bothered at all | 2634 | | (19.4) | | 486 | | (21.6) | 753 | (23.2) | 1354 | (17.1) | 41 | (27.9) |  |  |
| Bothered a little | 6598 | | (48.7) | | 1089 | | (48.5) | 1584 | (48.9) | 3864 | (48.8) | 61 | (41.5) |  |  |
| Bothered a lot | 4309 | | (31.8) | | 671 | | (29.9) | 902 | (27.8) | 2691 | (34.0) | 45 | (30.6) |  |  |
| Missing data | 3 | | (＜0.1) | | 0 | | (0) | 0 | (0) | 3 | (0.0) | 0 | (0) |  |  |
| **Pain in your arms, legs, or joints** | | | | | | | | |  |  |  |  |  |  | <0.001 |
| Not bothered at all | 1803 | | (13.3) | | 366 | | (16.3) | 501 | (15.5) | 907 | (11.5) | 29 | (19.7) |  |  |
| Bothered a little | 5997 | | (44.3) | | 1028 | | (45.8) | 1460 | (4.9) | 3439 | (43.5) | 70 | (47.6) |  |  |
| Bothered a lot | 5738 | | (42.4) | | 852 | | (37.9) | 1278 | (39.5) | 3560 | (45.0) | 48 | (32.7) |  |  |
| Missing data | 6 | | (＜0.1) | | 0 | | (0) | 0 | (0) | 6 | (0.1) | 0 | (0) |  |  |
| **Menstrual cramps or other problems with your periods** | | | | | | | | | | |  |  |  |  | 0.045 |
| Not bothered at all | 1700 | | (12.6) | | 316 | | (14.1) | 465 | (14.4) | 900 | (11.4) | 19 | (12.9) |  |  |
| Bothered a little | 618 | | (4.6) | | 109 | | (4.9) | 140 | (4.3) | 366 | (4.6) | 3 | (2.0) |  |  |
| Bothered a lot | 320 | | (2.4) | | 50 | | (2.2) | 76 | (2.3) | 192 | (2.4) | 2 | (1.4) |  |  |
| Missing data | 10906 | | (80.5) | | 1771 | | (78.9) | 2558 | (79.0) | 6454 | (81.6) | 123 | (83.7) |  |  |
| **Headaches** | | | | | | |  |  |  |  |  |  |  |  | <0.001 |
| Not bothered at all | 4708 | | (34.8) | | 804 | | (35.8) | 1311 | (40.5) | 2528 | (32.0) | 65 | (44.2) |  |  |
| Bothered a little | 6911 | | (51.0) | | 1136 | | (50.6) | 1541 | (47.6) | 4167 | (52.7) | 67 | (45.6) |  |  |
| Bothered a lot | 1920 | | (14.2) | | 306 | | (13.6) | 385 | (11.9) | 1214 | (15.3) | 15 | (10.2) |  |  |
| Missing data | 5 | | (＜0.1) | | 0 | | (0) | 2 | (0.1) | 3 | (0.0) | 0 | (0) |  |  |
| **Chest pain** | | | | | | |  |  |  |  |  |  |  |  | <0.001 |
| Not bothered at all | 9741 | | (71.9) | | 1677 | | (74.7) | 2469 | (76.2) | 5488 | (69.4) | 107 | (72.8) |  |  |
| Bothered a little | 3320 | | (24.5) | | 500 | | (22.3) | 679 | (21.0) | 2104 | (26.6) | 37 | (25.2) |  |  |
| Bothered a lot | 468 | | (3.5) | | 67 | | (3.0) | 87 | (2.7) | 311 | (3.9) | 3 | (2.0) |  |  |
| Missing data | 15 | | (＜0.1) | | 2 | | (0.1) | 4 | (0.1) | 9 | (0.1) | 0 | (0) |  |  |
| **Dizziness** |  | |  | |  | |  |  |  |  |  |  |  |  | <0.001 |
| Not bothered at all | 6935 | | (51.2) | | 1211 | | (53.9) | 1751 | (54.1) | 3891 | (49.2) | 82 | (55.8) |  |  |
| Bothered a little | 5699 | | (42.1) | | 919 | | (40.9) | 1286 | (39.7) | 3432 | (43.4) | 62 | (42.2) |  |  |
| Bothered a lot | 903 | | (6.7) | | 114 | | (5.1) | 199 | (6.1) | 587 | (7.4) | 3 | (2.0) |  |  |
| Missing data | 7 | | (＜0.1) | | 2 | | (0.1) | 3 | (0.1) | 2 | (0.0) | 0 | (0) |  |  |
| **Fainting spells** | | | | | | |  |  |  |  |  |  |  |  | 0.042 |
| Not bothered at all | 12569 | | (92.8) | | 2090 | | (93.1) | 3043 | (93.9) | 7303 | (92.3) | 133 | (90.5) |  |  |
| Bothered a little | 880 | | (6.5) | | 141 | | (6.3) | 175 | (5.4) | 550 | (7.0) | 14 | (9.5) |  |  |
| Bothered a lot | 71 | | (0.5) | | 13 | | (0.6) | 14 | (0.4) | 44 | (0.6) | 0 | (0) |  |  |
| Missing data | 24 | | (0.2) | | 2 | | (0.1) | 7 | (0.2) | 15 | (0.2) | 0 | (0) |  |  |
| **Feeling your heart pound or race** | | | | | | | | |  |  |  |  |  |  | <0.001 |
| Not bothered at all | 6182 | | (45.6) | | 1108 | | (49.3) | 1584 | (48.9) | 3410 | (43.1) | 80 | (54.4) |  |  |
| Bothered a little | 5962 | | (44.0) | | 925 | | (41.2) | 1341 | (41.4) | 3644 | (46.1) | 52 | (35.4) |  |  |
| Bothered a lot | 1394 | | (10.3) | | 213 | | (9.5) | 313 | (9.7) | 853 | (10.8) | 15 | (10.2) |  |  |
| Missing data | 6 | | (＜0.1) | | 0 | | (0) | 1 | (＜0.1) | 5 | (0.1) | 0 | (0) |  |  |
| **Shortness of breath** | | | | | | | |  |  |  |  |  |  |  | <0.001 |
| Not bothered at all | 7188 | | (53.1) | | 1319 | | (58.7) | 1777 | (54.9) | 4010 | (50.7) | 82 | (55.8) |  |  |
| Bothered a little | 5044 | | (37.2) | | 765 | | (34.1) | 1160 | (35.8) | 3067 | (38.8) | 52 | (35.4) |  |  |
| Bothered a lot | 1305 | | (9.6) | | 162 | | (7.2) | 297 | (9.2) | 833 | (10.5) | 13 | (8.8) |  |  |
| Missing data | 7 | | (＜0.1) | | 0 | | (0) | 5 | (0.2) | 2 | (＜0.1) | 0 | (0) |  |  |
| **Pain or problems during intercourse** | | | | | | | | |  |  |  |  |  |  | <0.001 |
| Not bothered at all | | 4044 | | (29.9) | 692 | (30.8) | | 1079 | (33.3) | 2228 | (28.2) | 45 | (30.6) |  |  |
| Bothered a little | | 2079 | | (15.3) | 361 | (16.1) | | 441 | (13.6) | 1259 | (15.9) | 18 | (12.2) |  |  |
| Bothered a lot | | 1197 | | (8.8) | 221 | (9.8) | | 241 | (7.4) | 726 | (9.2) | 9 | (6.1) |  |  |
| Missing data | | 6224 | | (46.0) | 972 | (43.3) | | 1478 | (45.6) | 3699 | (46.8) | 75 | (51.0) |  |  |
| **Feeling tired all the time or having low energy** | | | | | | | | | |  |  |  |  |  | <0.001 |
| Not bothered at all | 1412 | | (10.4) | | 292 | | (13.0) | 373 | (11.5) | 729 | (9.2) | 18 | (12.2) |  |  |
| Bothered a little | 5910 | | (43.6) | | 1045 | | (46.5) | 1451 | (44.8) | 3351 | (42.4) | 63 | (42.9) |  |  |
| Bothered a lot | 6217 | | (45.9) | | 909 | | (40.5) | 1414 | (43.7) | 3828 | (48.4) | 66 | (44.9) |  |  |
| Missing data | 5 | | (＜0.1) | | 0 | | (0) | 1 | (＜0.1) | 4 | (0.1) | 0 | (0) |  |  |
| **Trouble sleeping** | | | | | | |  |  |  |  |  |  |  |  | <0.001 |
| Not bothered at all | 1896 | | (14.0) | | 346 | | (15.4) | 530 | (16.4) | 985 | (12.4) | 35 | (23.8) |  |  |
| Bothered a little | 6006 | | (44.3) | | 1044 | | (46.5) | 1482 | (45.8) | 3421 | (43.2) | 59 | (40.1) |  |  |
| Bothered a lot | 5635 | | (41.6) | | 855 | | (38.1) | 1226 | (37.9) | 3501 | (44.2) | 53 | (36.1) |  |  |
| Missing data | 7 | | (＜0.1) | | 1 | | (＜0.1) | 1 | (＜0.1) | 5 | (0.1) | 0 | (0) |  |  |

The question for “menstrual cramps or other problems with the period” was taken into the analysis only in female sex. In this analysis, “Do not know”, “Prefer not to answer” and “missing” were coded as missing data.

Table S5. Logistic regression analysis of factors associated with IBS, stratified by sex

| **Variable** | **Model 1** | | | |  | **Model 2** | | | |
| --- | --- | --- | --- | --- | --- | --- | --- | --- | --- |
|  | **Men Odds ratio (95% confidence interval)** | **p value** | **Women Odds ratio (95% confidence interval)** | **p value** |  | **Men Odds ratio (95% confidence interval)** | **p value** | **Women Odds ratio (95% confidence interval)** | **p value** |
| **Age (years)** |  |  |  |  |  |  |  |  |  |
|  | NA |  | NA |  |  | 0.985  (0.979-0.990) | <0.001 | 0.985  (0.981-0.988) | <0.001 |
| **Townsend Deprivation Score** | | | | |  |  |  |  |  |
|  | NA |  | NA |  |  | 1.028  (1.013-1.044) | <0.001 | 0.996  (0.987-1.005) | 0.344 |
| **Ever been offered/sought treatment for anxiety** | | | |  |  |  |  |  |  |
|  | 1.438  (1.276-1.621) | <0.001 | 1.344  (1.263-1.429) | <0.001 |  | 1.406  (1.247-1.585) | <0.001 | 1.343  (1.263-1.429) | <0.001 |
| **Ever been offered/sought treatment for depression** | | | |  |  |  |  |  |  |
|  | 1.447  (1.286-1.628) | <0.001 | 1.302  (1.225-1.383) | <0.001 |  | 1.404  (1.248-1.581) | <0.001 | 1.281  (1.206-1.361) | <0.001 |
| **Family history of IBS** | | | |  |  |  |  |  |  |
|  | 3.847  (3.481-4.251) | <0.001 | 3.091  (2.928-3.263) | <0.001 |  | 3.789  (3.427-4.190) | <0.001 | 3.054  (2.893-3.224) | <0.001 |
| **Long-term/recurrent antibiotics as child or teenager** | | | |  |  |  |  |  |  |
|  | 1.830  (1.628-2.056) | <0.001 | 1.690  (1.594-1.792) | <0.001 |  | 1.758  (1.563-1.977) | <0.001 | 1.649  (1.555-1.749) | <0.001 |
| **Diagnosed with coeliac disease or gluten sensitivity** | | | |  |  |  |  |  |  |
|  | 4.086  (3.121-5.349) | <0.001 | 3.779  (3.308-4.317) | <0.001 |  | 4.107  (3.132-5.385) | <0.001 | 3.783  (3.310-4.323) | <0.001 |
| **PHQ-12 score** |  |  |  |  |  |  |  |  |  |
|  | 5.372  (1.907-5.881) | <0.001 | 4.786  (4.544-5.041) | <0.001 |  | 5.326  (4.863-5.832) | <0.001 | 4.738  (4.498-4.992) | <0.001 |

NA=not applicable; Model 1: No adjustment; Model 2: Adjustment for Age and Townsend Deprivation Score; Missing data were excluded in this analysis.

Table S6. Multiple logistic regression analysis of potential influencing factors for different IBS subtypes stratified by sex

| **Variable** | **Model 3** | | | |  | **Model 4** | | | |
| --- | --- | --- | --- | --- | --- | --- | --- | --- | --- |
|  | **Men** | **p-value** | **Women** | **p-value** |  | **Men** | **p-value** | **Women** | **p-value** |
|  | **OR (95%CI)** |  | **OR (95%CI)** |  |  | **OR (95%CI)** |  | **OR (95%CI)** |  |
| **Age** | | | | | | | | | |
| IBS-C | NA |  | NA |  |  | 1.006 | 0.544 | 0.980 | ＜0.001 |
|  | NA |  | NA |  |  | (0.988,1.024) |  | (0.973,0.987) |  |
| IBS-D | NA |  | NA |  |  | 0.970 | ＜0.001 | 0.986 | ＜0.001 |
|  | NA |  | NA |  |  | (0.961,0.979) |  | (0.980,0.992) |  |
| IBS-M | NA |  | NA |  |  | 0.988 | 0.002 | 0.984 | ＜0.001 |
|  | NA |  | NA |  |  | (0.981,0.996) |  | (0.980,0.988) |  |
| IBS-U | NA |  | NA |  |  | 1.044 | 0.089 | 1.034 | 0.016 |
|  | NA |  | NA |  |  | (0.994,1.097) |  | (1.006,1.063) |  |
| **Townsend Deprivation Score** | | | | | | | | | |
| IBS-C | NA |  | NA |  |  | 1.061 | 0.007 | 0.982 | 0.067 |
|  | NA |  | NA |  |  | (1.016,1.109) |  | (0.964,1.001) |  |
| IBS-D | NA |  | NA |  |  | 1.007 | 0.619 | 0.997 | 0.734 |
|  | NA |  | NA |  |  | (0.981,1.033) |  | (0.982,1.013) |  |
| IBS-M | NA |  | NA |  |  | 1.034 | ＜0.001 | 0.999 | 0.918 |
|  | NA |  | NA |  |  | (1.015,1.054) |  | (0.989,1.010) |  |
| IBS-U | NA |  | NA |  |  | 1.023 | 0.720 | 0.977 | 0.533 |
|  | NA |  | NA |  |  | (0.905,1.155) |  | (0.908,1.051) |  |
| **Ever been offered/sought treatment for anxiety** | | | | | | | | | |
| IBS-C | 1.605 | 0.010 | 1.234 | 0.002 |  | 1.581 | 0.013 | 1.237 | 0.002 |
|  | (1.122,2.296) |  | (1.081,1.407) |  |  | (1.102,2.266) |  | (1.084,1.411) |  |
| IBS-D | 1.449 | ＜0.001 | 1.365 | ＜0.001 |  | 1.406 | 0.001 | 1.363 | ＜0.001 |
|  | (1.181,1.777) |  | (1.224,1.523) |  |  | (1.146,1.725) |  | (1.222,1.520) |  |
| IBS-M | 1.430 | ＜0.001 | 1.361 | ＜0.001 |  | 1.401 | ＜0.001 | 1.361 | ＜0.001 |
|  | (1.229,1.663) |  | (1.261,1.470) |  |  | (1.204,1.630) |  | (1.260,1.469) |  |
| IBS-U | 0.624 | 0.404 | 1.755 | 0.023 |  | 0.637 | 0.426 | 1.770 | 0.022 |
|  | (0.206,1.889) |  | (1.081,2.850) |  |  | (0.209,1.937) |  | (1.088,2.882) |  |
| **Ever been offered/sought treatment for depression** | | | | | | | | | |
| IBS-C | 1.452 | 0.040 | 1.277 | ＜0.001 |  | 1.396 | 0.070 | 1.254 | 0.001 |
|  | (1.017,2.073) |  | (1.123,1.452) |  |  | (0.974,2.000) |  | (1.102,1.426) |  |
| IBS-D | 1.246 | 0.034 | 1.279 | ＜0.001 |  | 1.203 | 0.076 | 1.262 | ＜0.001 |
|  | (1.016,1.528) |  | (1.149,1.424) |  |  | (0.981,1.476) |  | (1.133,1.406) |  |
| IBS-M | 1.555 | ＜0.001 | 1.329 | ＜0.001 |  | 1.514 | ＜0.001 | 1.306 | ＜0.001 |
|  | (1.341,1.803) |  | (1.233,1.434) |  |  | (1.305,1.757) |  | (1.211,1.409) |  |
| IBS-U | 1.763 | 0.235 | 0.920 | 0.740 |  | 1.856 | 0.200 | 0.957 | 0.863 |
|  | (0.691,4.499) |  | (0.560,1.509) |  |  | (0.721,4.778) |  | (0.581,1.576) |  |
| **Family history of IBS** | | | | | | | | | |
| IBS-C | 2.527 | ＜0.001 | 2.703 | ＜0.001 |  | 2.579 | ＜0.001 | 2.659 | ＜0.001 |
|  | (1.830,3.490) |  | (2.412,3.028) |  |  | (1.865,3.566) |  | (2.372,2.980) |  |
| IBS-D | 4.016 | ＜0.001 | 2.839 | ＜0.001 |  | 3.874 | ＜0.001 | 2.813 | ＜0.001 |
|  | (3.407,4.735) |  | (2.582,3.121) |  |  | (3.283,4.570) |  | (2.558,3.093) |  |
| IBS-M | 4.034 | ＜0.001 | 3.389 | ＜0.001 |  | 3.992 | ＜0.001 | 3.348 | ＜0.001 |
|  | (3.559,4.571) |  | (3.172,3.622) |  |  | (3.520,4.527) |  | (3.132,3.578) |  |
| IBS-U | 2.660 | 0.024 | 1.345 | 0.235 |  | 2.827 | 0.017 | 1.374 | 0.204 |
|  | (1.139,6.215) |  | (0.824,2.194) |  |  | (1.206,6.625) |  | (0.842,2.243) |  |
| **Long-term/recurrent antibiotics as child or teenager** | | | | | | | | | |
| IBS-C | 2.055 | ＜0.001 | 1.578 | ＜0.001 |  | 2.065 | ＜0.001 | 1.534 | <0.001 |
|  | (1.467,2.879) |  | (1.395,1.784) |  |  | (1.471,2.900) |  | (1.355,1.735) |  |
| IBS-D | 1.767 | ＜0.001 | 1.568 | ＜0.001 |  | 1.660 | ＜0.001 | 1.528 | ＜0.001 |
|  | (1.453,2.150) |  | (1.414,1.738) |  |  | (1.363,2.022) |  | (1.378,1.696) |  |
| IBS-M | 1.843 | ＜0.001 | 1.778 | ＜0.001 |  | 1.775 | ＜0.001 | 1.736 | ＜0.001 |
|  | (1.593,2.132) |  | (1.656,1.910) |  |  | (1.532,2.057) |  | (1.615,1.865) |  |
| IBS-U | 1.216 | 0.718 | 1.851 | 0.007 |  | 1.317 | 0.613 | 1.979 | 0.003 |
|  | (0.421,3.516) |  | (1.182,2.901) |  |  | (0.453,3.829) |  | (1.259,3.111) |  |
| **Diagnosed with coeliac disease or gluten sensitivity** | | | | | | | | | |
| IBS-C | 3.610 | 0.001 | 3.966 | ＜0.001 |  | 3.672 | 0.001 | 3.991 | <0.001 |
|  | (1.674,7.788) |  | (3.128,5.028) |  |  | (1.702,7.924) |  | (3.147,5.061) |  |
| IBS-D | 4.446 | ＜0.001 | 3.995 | ＜0.001 |  | 4.351 | ＜0.001 | 4.005 | ＜0.001 |
|  | (2.969,6.657) |  | (3.264,4.889) |  |  | (2.885,6.562) |  | (3.272,4.903) |  |
| IBS-M | 3.978 | ＜0.001 | 3.581 | ＜0.001 |  | 4.054 | ＜0.001 | 3.576 | ＜0.001 |
|  | (2.859,5.536) |  | (3.056,4.197) |  |  | (2.912,5.644) |  | (3.050,4.193) |  |
| IBS-U | 3.768 | 0.193 | 5.699 | ＜0.001 |  | 3.723 | 0.197 | 5.677 | ＜0.001 |
|  | (0.511,27.810) |  | (2.742,11.842) |  |  | (0.504,27.471) |  | (2.730,11.807) |  |
| **PHQ-12>6** | | | | | | | | | |
| IBS-C | 4.363 | ＜0.001 | 4.211 | ＜0.001 |  | 4.334 | ＜0.001 | 4.173 | <0.001 |
|  | (3.302,5.766) |  | (3.761,4.715) |  |  | (3.275,5.735) |  | (3.726,4.673) |  |
| IBS-D | 4.757 | ＜0.001 | 3.769 | ＜0.001 |  | 4.726 | ＜0.001 | 3.736 | ＜0.001 |
|  | (4.075,5.554) |  | (3.434,4.138) |  |  | (4.046,5.520) |  | (3.403,4.103) |  |
| IBS-M | 5.976 | ＜0.001 | 5.596 | ＜0.001 |  | 5.915 | ＜0.001 | 5.531 | ＜0.001 |
|  | (5.317,6.717) |  | (5.229,5.989) |  |  | (5.260,6.651) |  | (5.166,5.920) |  |
| IBS-U | 4.344 | ＜0.001 | 4.540 | ＜0.001 |  | 4.314 | ＜0.001 | 4.676 | ＜0.001 |
|  | (2.102,8.976) |  | (2.950,6.987) |  |  | (2.085,8.925) |  | (3.037,7.199) |  |

Model 3: No adjustment; Model 4: Adjustment for Age and Townsend Deprivation Score; NA: Not appliable. Missing data were excluded in this analysis.

Table S7. Chi-Square Analysis of Differences in Influencing Factors among Different Subtypes in males (n = 4151)

|  | **IBS-C** | | | **IBS-D** | | | **IBS-M** | | | **IBS-U** | | | **p-value** |
| --- | --- | --- | --- | --- | --- | --- | --- | --- | --- | --- | --- | --- | --- |
|  | **n= 362** | | | **n= 1209** | | | **n= 2524** | | | **n= 56** | | |  |
| **Age (years)** | |  | |  | | |  | | |  | | | ＜0.001 |
| Mean (SD) | 56.35 | | (7.981) | 54.30 | (7.961) | | 55.1 | | (7.978) | 58.41 | (7.478) | |  |
| Median (IQR) | 58 | | (50,63) | 55 | (47.5,61) | | 56 | | (48,62) | 60 | (53,64) | |  |
| **Townsend Deprivation Score** | | | |  | | |  | | |  | | | 0.178 |
| Mean (SD) | -1.16 | | (3.175) | -1.48 | | (3.041) | -1.22 | (3.203) | | -1.46 | | (3.214) |  |
| Median (IQR) | -1.87 | | (-3.79,0.58) | -2.29 | | (-3.78,0.33) | -2.14 | (-3.68,0.76) | | -2.60 | | (-3.81,0.31) |  |
| Missing data | 1 | | (0.3) | 2 | | (0.2) | 6 | (0.2) | | 1 | | (1.8) |  |
| **Ever been offered/sought treatment for anxiety** | | | | | | | | | | | | | 0.163 |
| Yes | 107 | | (29.6) | 396 | | (32.8) | 852 | (33.8) | | 13 | | (23.2) |  |
| No | 254 | | (70.2) | 811 | | (67.1) | 1664 | (65.9) | | 43 | | (76.8) |  |
| Missing data | 1 | | (0.3) | 2 | | (0.2) | 8 | (0.3) | | 0 | | (0) |  |
| **Ever been offered/sought treatment for depression** | | | | | | | | | | | | | 0.041 |
| Yes | 118 | | (32.6) | 377 | | (31.2) | 894 | (35.4) | | 15 | | (26.8) |  |
| No | 243 | | (67.1) | 830 | | (68.7) | 1619 | (64.1) | | 40 | | (71.4) |  |
| Missing data | 1 | | (0.3) | 2 | | (0.2) | 11 | (0.4) | | 1 | | (1.8) |  |
| **Family history of IBS** | | | | | | | | | | | | | 0.010 |
| Yes | 65 | | (18.0) | 277 | | (22.9) | 590 | (23.4) | | 9 | | (16.1) |  |
| No | 189 | | (52.2) | 556 | | (46.0) | 1075 | (42.6) | | 28 | | (50.0) |  |
| Missing data | 108 | | (29.8) | 376 | | (31.1) | 859 | (34.0) | | 19 | | (33.9) |  |
| **Long-term/recurrent antibiotics as child or teenager** | | | | | | | | | | | | | 0.333 |
| Yes | 66 | | (18.2) | 193 | | (16.0) | 447 | (17.7) | | 9 | | (16.1) |  |
| No | 240 | | (66.3) | 842 | | (69.6) | 1649 | (65.3) | | 40 | | (71.4) |  |
| Missing data | 56 | | (15.5) | 174 | | (14.4) | 428 | (17.0) | | 7 | | (12.5) |  |
| **Diagnosed with coeliac disease or gluten sensitivity** | | | | | | | | | | | | | 0.574 |
| Yes | 11 | | (3.0) | 38 | | (3.1) | 96 | (3.8) | | 1 | | (1.8) |  |
| No | 337 | | (93.1) | 1124 | | (93.0) | 2312 | (91.6) | | 54 | | (96.4) |  |
| Missing data | 14 | | (3.9) | 47 | | (3.9) | 116 | (4.6) | | 1 | | (1.8) |  |
| **PHQ-12 score** | | | | | | | | | | | | | ＜0.001 |
| ≤ 6 | 158 | | (43.6) | 571 | | (47.2) | 941 | (37.3) | | 28 | | (50.0) |  |
| > 6 | 185 | | (51.1) | 599 | | (49.5) | 1448 | (57.4) | | 24 | | (42.9) |  |
| Missing data | 19 | | (5.2) | 39 | | (3.2) | 135 | (5.3) | | 4 | | (7.1) |  |

Data were mean (SD) or n (%) unless noted otherwise. The distribution of age and Townsend Deprivation Score is non-normal, therefore, the mean (SD) and median (P25,P75) are used to describe. The p value was calculated by the chi-square test and Wilcoxon’s rank-sum test where applicable. In this analysis, “Do not know”, “Prefer not to answer” and “missing” were coded as missing data.

Table S8. Chi-Square Analysis of Differences in Influencing Factors among Different Subtypes in females (n = 13544)

|  | **IBS-C** | | **IBS-D** | | **IBS-M** | | **IBS-U** | | **p-value** |
| --- | --- | --- | --- | --- | --- | --- | --- | --- | --- |
|  | **n= 2246** | | **n= 3239** | | **n= 7912** | | **n= 147** | |  |
| **Age (years)** | | | | | | | | | 0.001 |
| Mean (SD) | 54.09 | (7.795) | 54.25 | (7.693) | 54.11 | (7.711) | 56.76 | (7.461) |  |
| Median (IQR) | 54 | (48,60) | 54 | (48,61) | 54 | (48,60) | 58 | (51,63) |  |
| **Townsend Deprivation Score** | | | | | | | | | 0.009 |
| Mean (SD) | -1.64 | (2.850) | -1.47 | (2.907) | -1.39 | (3.016) | -1.95 | (2.485) |  |
| Median (IQR) | -2.38 | (-3.76,-0.12) | -2.26 | (-3.69,0.29) | -2.19 | (-3.67,0.44) | -3.88 | (-2.40,-0.76) |  |
| Missing data | 3 | (0.1) | 5 | (0.2) | 11 | (0.1) | 1 | (0.7) |  |
| **Ever been offered/sought treatment for anxiety** | | | | | | | | | 0.002 |
| Yes | 824 | (36.7) | 1232 | (38.0) | 3207 | (40.5) | 61 | (41.5) |  |
| No | 1413 | (62.9) | 2002 | (61.8) | 4662 | (58.9) | 86 | (58.5) |  |
| Missing data | 9 | (0.4) | 5 | (0.2) | 43 | (0.5) | 0 | (0) |  |
| **Ever been offered/sought treatment for depression** | | | | | | | | | ＜0.001 |
| Yes | 908 | (40.4) | 1336 | (41.2) | 3527 | (44.6) | 53 | (36.1) |  |
| No | 1325 | (59.0) | 1891 | (58.4) | 4350 | (55.0) | 93 | (63.3) |  |
| Missing data | 13 | (0.6) | 12 | (0.4) | 35 | (0.4) | 1 | (0.7) |  |
| **Family history of IBS** | | | | | | | | | ＜0.001 |
| Yes | 621 | (27.6) | 946 | (29.2) | 2458 | (31.1) | 27 | (18.4) |  |
| No | 1080 | (48.1) | 1526 | (47.1) | 3322 | (42.0) | 83 | (56.5) |  |
| Missing data | 545 | (24.3) | 767 | (23.7) | 2132 | (26.9) | 37 | (25.2) |  |
| **Long-term/recurrent antibiotics as child or teenager** | | | | | | | | | ＜0.001 |
| Yes | 521 | (23.2) | 775 | (23.9) | 2115 | (26.7) | 37 | (25.2) |  |
| No | 1412 | (62.9) | 2060 | (63.6) | 4667 | (59.0) | 93 | (63.3) |  |
| Missing data | 313 | (13.9) | 404 | (12.5) | 1130 | (14.3) | 17 | (11.6) |  |
| **Diagnosed with coeliac disease or gluten sensitivity** | | | | | | | | | 0.730 |
| Yes | 122 | (5.4) | 184 | (5.7) | 427 | (5.4) | 11 | (7.5) |  |
| No | 2071 | (92.2) | 2999 | (92.6) | 7318 | (92.5) | 136 | (92.5) |  |
| Missing data | 53 | (2.4) | 56 | (1.7) | 167 | (2.1) | 0 | (0) |  |
| **PHQ-12 score** | | | | | | | | | ＜0.001 |
| ≤ 6 | 770 | (34.3) | 1154 | (35.6) | 2071 | (26.2) | 55 | (37.4) |  |
| > 6 | 1388 | (61.8) | 1956 | (60.4) | 5485 | (69.3) | 89 | (60.5) |  |
| Missing data | 88 | (3.9) | 129 | (4.0) | 356 | (4.5) | 3 | (2.0) |  |

Data were mean (SD) or n (%) unless noted otherwise. The distribution of age and Townsend Deprivation Score is non-normal, therefore, the mean (SD) and median (P25,P75) are used to describe. The p value was calculated by the chi-square test and Wilcoxon’s rank-sum test where applicable. In this analysis, “Do not know”, “Prefer not to answer” and “missing” were coded as missing data.
